# Cuproptosis-related gene PROK1 predicts the diagnosis and prognosis of prostate cancer based on multiple machine learning

**DOI:** 10.7150/jca.113505

**Published:** 2026-01-01

**Authors:** Xin Qin, Qinghua Wang, Wei Jiang, Yan Zhao, Haopeng Li, Tong Zi, Yaru Zhu, Xilei Li, Chengdang Xu, Tao Yang, Xinan Wang, Yicong Yao, Xi Chen, Juan Zhou, Gang Wu

**Affiliations:** 1 Department of Urology, Tongji Hospital, School of Medicine, Tongji University, Shanghai 200092, China.; 2 ICU, Tongji Hospital, School of Medicine, Tongji University, Shanghai 200092, China.

**Keywords:** cuproptosis, prostate cancer, tumor immune microenvironment, machine learning, diagnosis, prognosis, PROK1

## Abstract

Cuproptosis, a newly identified form of cell death, influences the development, progression, and prognosis of prostate cancer (PCa). Identifying key genes associated with cuproptosis and developing robust predictive models through machine learning approaches are crucial for personalized PCa treatment. In our study, multiple machine learning methods and their combinations were employed for the construction of diagnostic and prognostic models for PCa, which were then validated in multiple external independent cohorts. The model key gene, PROK1, was selected for further analysis, and its expression was compared in clinical samples and cell lines. Additionally, the anticancer effect of PROK1 was explored through regulating the expression of PROK1. Most cuproptosis-related genes (CRGs) showed differential expression between PCa and normal prostate tissues. The two clusters derived from the Consensus Clustering method, based on cuproptosis gene expression characteristics, exhibit distinct clinical features and immune microenvironment infiltration patterns. Models constructed based on machine learning methods showed promising diagnostic capabilities for PCa and were associated with the prediction of biochemical recurrence-free survival and disease-free survival of patients. Inhibiting PROK1 expression promoted PCa cell proliferation and invasion, while its overexpression had the opposite effect. Furthermore, pathway exploration revealed that PROK1 inhibits tumor growth by mediating apoptosis under copper ion stress. Its association with cuproptosis warrants further investigation to elucidate the precise mechanism.

## 1. Introduction

Prostate cancer (PCa) is a common malignancy of the genitourinary system in elderly men worldwide, with its incidence increasing with age, particularly in men over 65 years old^1^. According to 2023 statistics from the United States, PCa ranks first in the number of new tumor cases in men, accounting for 29% of cases, and has the second highest mortality rate at 11%^2^. Currently, the early diagnosis and prognosis assessment of PCa mainly rely on the Gleason score, TNM stage, prostate-specific antigen (PSA) level, surgical margin, etc., all of which demonstrate commendable predictive ability. However, PCa exhibits significant heterogeneity^3^. For this reason, more sensitive biomarkers need to be developed to better classify the PCa population and better implement treatment^4^.

Copper is a metal ion with redox activity and can participate in a variety of biological processes, such as regulating energy production and other redox reactions, as well as the metabolism of glucose, cholesterol, and iron^5^. It often acts as a structural or catalytic cofactor of enzymes^6^. Recent studies have shown that copper-dependent cell death can occur by directly binding to lipoylated components of the tricarboxylic acid (TCA) cycle. Mechanistically, cell death caused by copper ions may involve the accumulation of lipoylated proteins, loss of iron-sulfur cluster proteins, and subsequent proteotoxicity^7^. Research has indicated that disorders of copper metabolism in organisms can result in nervous system diseases^8^, ^9^. Copper ions have been found to be closely related to immunity, for example, a deficiency in copper affects the number of neutrophils in the blood, which in turn affects the body's immune system^10^. Immunotherapy plays an important role in many advanced cancers, with the exception of PCa^11^, ^12^. Many previous studies have explored the relationship between cuproptosis and a variety of cancers at the genetic level, such as liver cancer, breast cancer, renal clear cell carcinoma, etc.^13^-^15^. Research indicates that cuproptosis may impact the prognosis of PCa. Using genes such as PRLR, DES, and LECT2, researchers have developed a prognostic model^16^. In addition, studies have successfully established a risk prediction model based on five copper-related genes: ATP7B, DBT, LIPT1, GCSH, and PDHA1, achieving promising predictive outcomes^17^. However, elucidating how cuproptosis and its associated genes influence PCa progression, and exploring more effective methods to construct robust predictive models, are areas deserving further investigation.

As medical understanding advances, vast amounts of medical record and gene sequencing data have emerged, ushering medicine into the era of big data^18^. Processing and mining this massive data requires advanced modern computing technology. Machine learning, a rapidly growing technology for integrating and analyzing complex data, can learn from this data and interpret unknown situations, and is increasingly applied in disease detection, diagnosis, and treatment^19^-^21^. Machine learning is well-suited for extracting key information from exponentially growing genomic data, efficiently exploiting large datasets, and has become the preferred method for many genomics models^22^.

PROK1, a member of the prokineticin family and also referred to as endocrine gland-derived vascular endothelial growth factor (EG-VEGF), is a vascular endothelial growth factor specifically expressed in endocrine glands^1^. PROK1 has been demonstrated to promote neovascularization and enhance vascular permeability by stimulating the proliferation of capillary endothelial cells within endocrine tissues. Both PROK1 and PROK2 activate two specific G-protein-coupled receptors: prokineticin receptor 1 (PKR1) and prokineticin receptor 2 (PKR2). Upon activation, these receptors trigger the mitogen-activated protein kinase (MAPK) and phosphatidylinositol 3-kinase (PI3K)/Akt pathways, thereby promoting cell proliferation and angiogenesis^23^, ^24^. PROK1 has been implicated in various malignancies, including pancreatic, colon, and ovarian cancers^25^-^27^. Nevertheless, the role of PROK1 in PCa remains unclear and warrants further investigation.

## 2. Results

### 2.1 The Landscape of cuproptosis-related genes (CRGs) in PCa reveals distinct expression patterns

**Figure 1:**
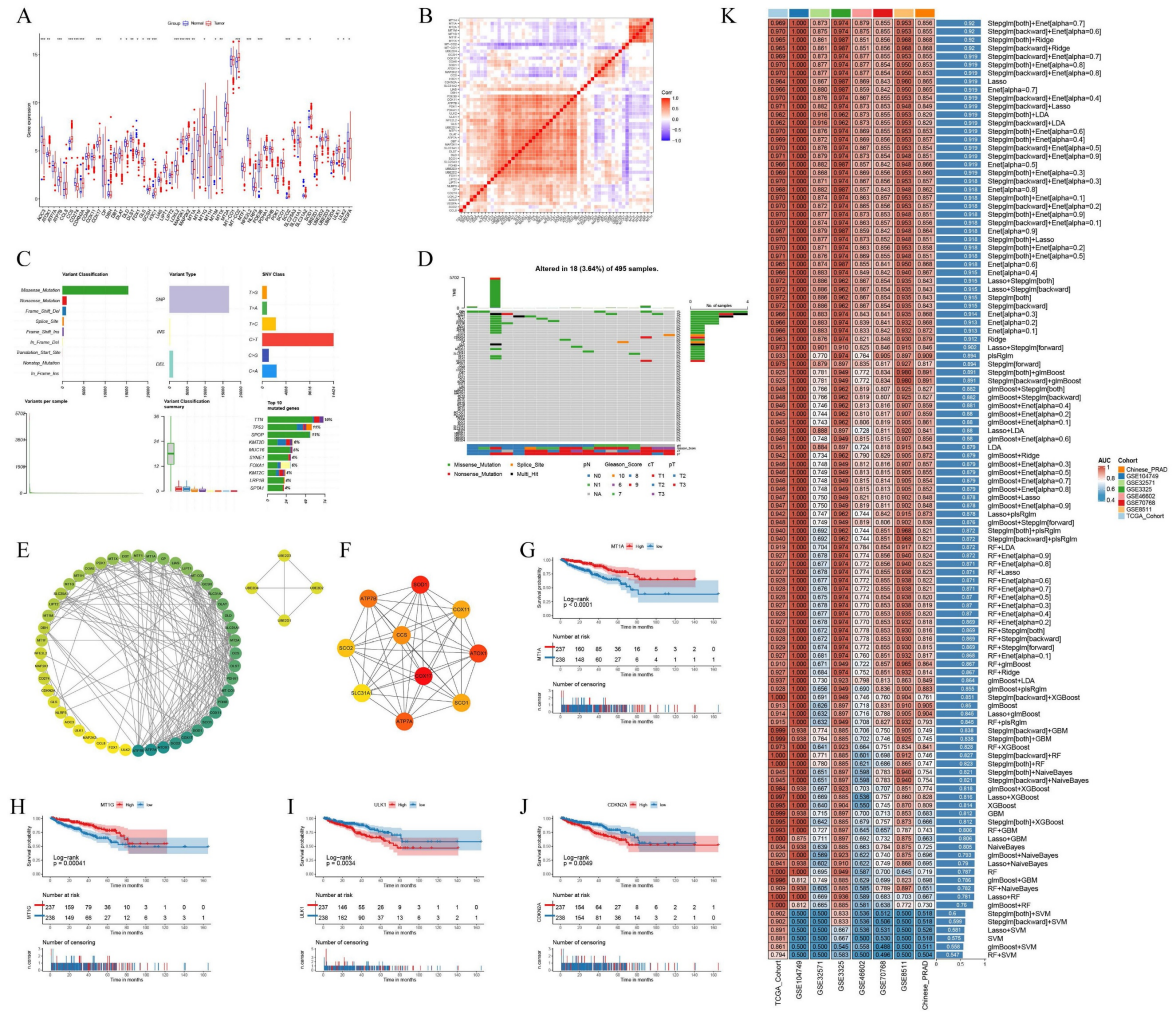
CRGs show a different landscape of expression, mutation and prognosis in PCa. (A) Expression of CRGs in normal and tumor samples. (B) Heat map of expression between CRGs. (C-D) Mutation frequency and mutation type of CRGs in PCa. (E-F) PPI regulatory networks of CRGs and hub genes. (G-J) Expression of CRGs and survival curves of DFS in PCa patients. (K) Multiple machine learning predictive models constructed based on the expression of CRGs. * *p*<0.05, ** *p*<0.01, *** *p*<0.001.

We analyzed the expression levels of 56 CRGs in PCa and tumor-adjacent tissues, identifying 30 differentially expressed CRGs. The expression of CRGs, including ATOX1, ATP7B, CCS, and CDKN2A, was upregulated in tumors, whereas the expression of AOC3 and CD274 was downregulated (Fig. 1A). Most CRGs exhibit co-expression relationships, suggesting complex coordination in biological processes (Fig. 1B). Missense mutations are the most prevalent type in the TCGA PCa cohort. Among the 56 CRGs, DBH, NLRP3, and MTF1 exhibit the highest mutation frequencies (Fig. 1C-D). Protein interaction data for 56 CRGs were retrieved from the STRING database and visualized using Cytoscape software. The circular protein-protein interaction (PPI) network, sorted by degree, reveals complex regulatory relationships among CRGs. Using the Cytohubba plug-in, we identified the top 10 hub genes according to the MCC method (Fig. 1E-F). Further analysis reveals that 16 CRGs may impact disease-free survival (DFS) in PCa patients (Fig. 1G-J, Fig. S1A-M).

Using the TCGA PCa cohort, multiple machine learning models were employed to assess the diagnostic value of CRGs, which were then validated against research cohorts from the GEO dataset and the Chinese PCa database. The Stepglm[both]+Enet[alpha=0.7] model showed the highest diagnostic performance, with an AUC value exceeding 0.85 in both the training and validation cohorts (Fig. 1K). The differential expression landscape of CRGs in PCa suggests a significant role in prostate carcinogenesis and patient outcomes modulation.

### 2.2 Clusters based on CRGs exhibited distinct prognostic features

**Figure 2:**
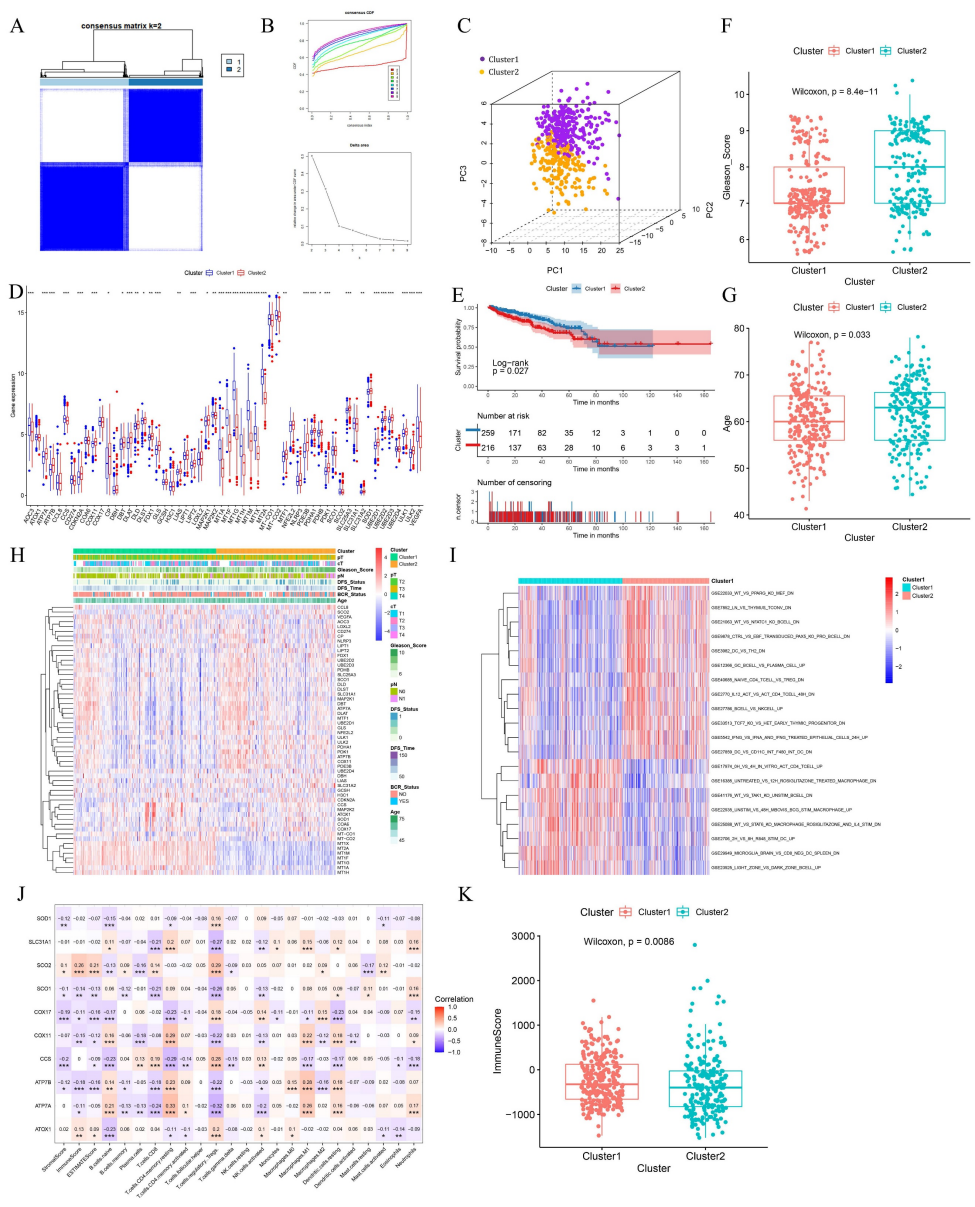
Clusters based on CRGs showed different prognostic manifestations. (A-B) Consensus matrix heatmap when k = 2 and consensus CDF when k = 2-9. (C) The three-dimensional principal component analysis scatter plots of two clusters. (D) Expression difference of CRGs between two clusters. (E) Survival analysis of the two clusters. (F-G) Differences in clinical features between the two clusters. (H) Heat maps of clinical information and gene expression between the two clusters. (I) Heat map of GSVA analysis results between the two clusters. (J) Heatmap of the correlation between hub genes and immunity scores, abundance of immune cell infiltration. (K) Differences in immunological scores between the two clusters. * *p*<0.05, ** *p*<0.01, *** *p*<0.001.

Using the R package ConsensusClusterPlus for consensus clustering analysis, we found that the cumulative distribution function (CDF) curve was smoother at k=2. The enhancement effect under the CDF curve was less pronounced at k=3, leading us to categorize PCa patients into two clusters (Fig. 2A-B). The clustering results were visualized using Principal Component Analysis (PCA), which demonstrated effective clustering (Fig. 2C). Most CRGs exhibited differential expression levels between the two clusters, indirectly indicating effective clustering (Fig. 2D). Comparative analysis of patient survival between the two clusters revealed that cluster 1 patients have significantly better survival than cluster 2 patients (*p*<0.05) (Fig. 2E). Cluster 1 patients had significantly lower Gleason scores and were younger than those in cluster 2 (Fig. 2F-G). Differences in clinical profiles and CRGs between the two subgroups were visually demonstrated using heat maps (Fig. 2H).

PCa is a typical immune cold tumor, and elucidating its enigmatic immune microenvironment is crucial for effective anti-tumor therapy. Immune-related gene sets were downloaded from the MsigDB database, and the Gene Set Variation Analysis (GSVA) algorithm was used to calculate the score for each patient. CRG-based clustering showed differences in the enrichment of immune-related gene sets, suggesting that CRGs may influence tumor development by affecting the PCa immune microenvironment (Fig. 2I). The expression of 10 hub genes was significantly correlated with the abundance of various tumor-infiltrating immune cells (Fig. 2J). Cluster 1 patients had significantly higher immune scores than cluster 2, suggesting that the high abundance of tumor-infiltrating immune cells may contribute to their better survival (Fig. 2K).

### 2.3 Prognostic modeling of DFS based on CRGs

**Figure 3:**
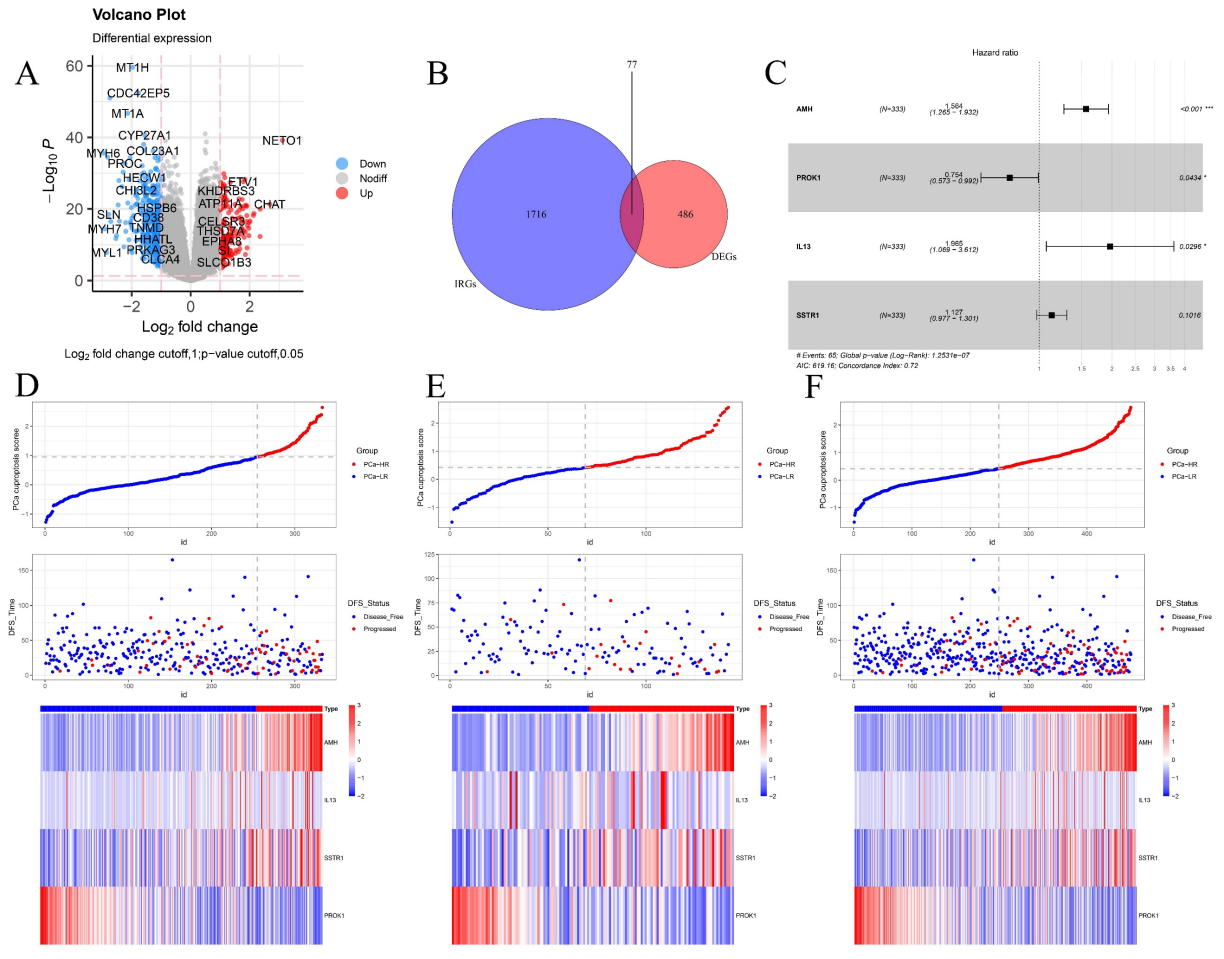
Constructing a cuproptosis-related DFS prognostic signature. (A) The volcano plot shows the differentially expressed genes between the two clusters. (B) The Venn diagram shows the intersection of cuproptosis related differentially expressed genes and immune-related genes. (C) The forest plot of the multivariable Cox regression analysis displays the four genes constituting the prognostic signature: AMH, PROK1, IL13, and SSTR1. (D-F) PCa cuproptosis score and expression levels of key genes between different risk groups in training set (D), internal validation set (E) and TCGA overall validation set (F) were demonstrated. * *p*<0.05, ** *p*<0.01, *** *p*<0.001.

Given that clustering based on CRGs shows differences in clinical phenotype, survival, and immune microenvironment, we performed differential expression analysis of the two clusters, selecting genes with |log2FC| > 1 and *p* < 0.05 for further study (Fig. 3A). These selected differential genes were intersected with immune-related genes and visualized in a Venn diagram (Fig. 3B). Patients in the TCGA cohort were divided into training and validation cohorts in a 7:3 ratio. Univariate COX regression analysis was initially used to screen genes related to DFS. In order to include more genes related to prognosis, the p-value was relaxed as permitted by the statistical method, and p < 0.2 was selected as the cut-off value to select differential genes. Next, the randomSurvivalForest package was used with 1000 trees for random forest analysis including survival information. The most important genes were identified using a cut-off value of 0.3 for gene importance. Stepwise regression was then used to retain genes with significant associations to survival outcomes, constructing the best signatures. Variables were screened based on the minimum AIC (Akaike Information Criterion) to prevent overfitting. Four genes, AMH, PROK1, IL13, and SSTR1, were ultimately selected and included in the multivariate regression analysis to construct the DFS prognostic prediction model (Fig. S2A-B). The PCa cuproptosis score was calculated using the following equation.



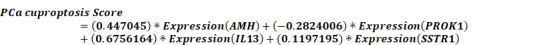



In the model, AMH, IL13, and SSTR1 were risk factors with hazard ratios (HR) > 1, while PROK1 was a protective factor with HR < 1 (Fig. 3C). Using the model formulation, we calculated the PCa cuproptosis score for each patient in the training and validation cohorts. Patients were categorized into PCa-high-risk (PCa-HR) and PCa-low risk (PCa-LR) groups using the optimal cut-off value calculated by the surv_cutpoint function of the survminer package. Scatter plots of the training cohort (Fig. 3D), validation cohort (Fig. 3E), and overall TCGA cohort (Fig. 3F) indicated higher PCa cuproptosis scores in patients with disease progression. Expression heatmaps showed increased levels of AMH, IL13, and SSTR1 and decreased levels of PROK1 in the PCa-HR group.

### 2.4 Differences in prognosis, clinical information, and immune microenvironment between PCa-HR and PCa-LR

**Figure 4:**
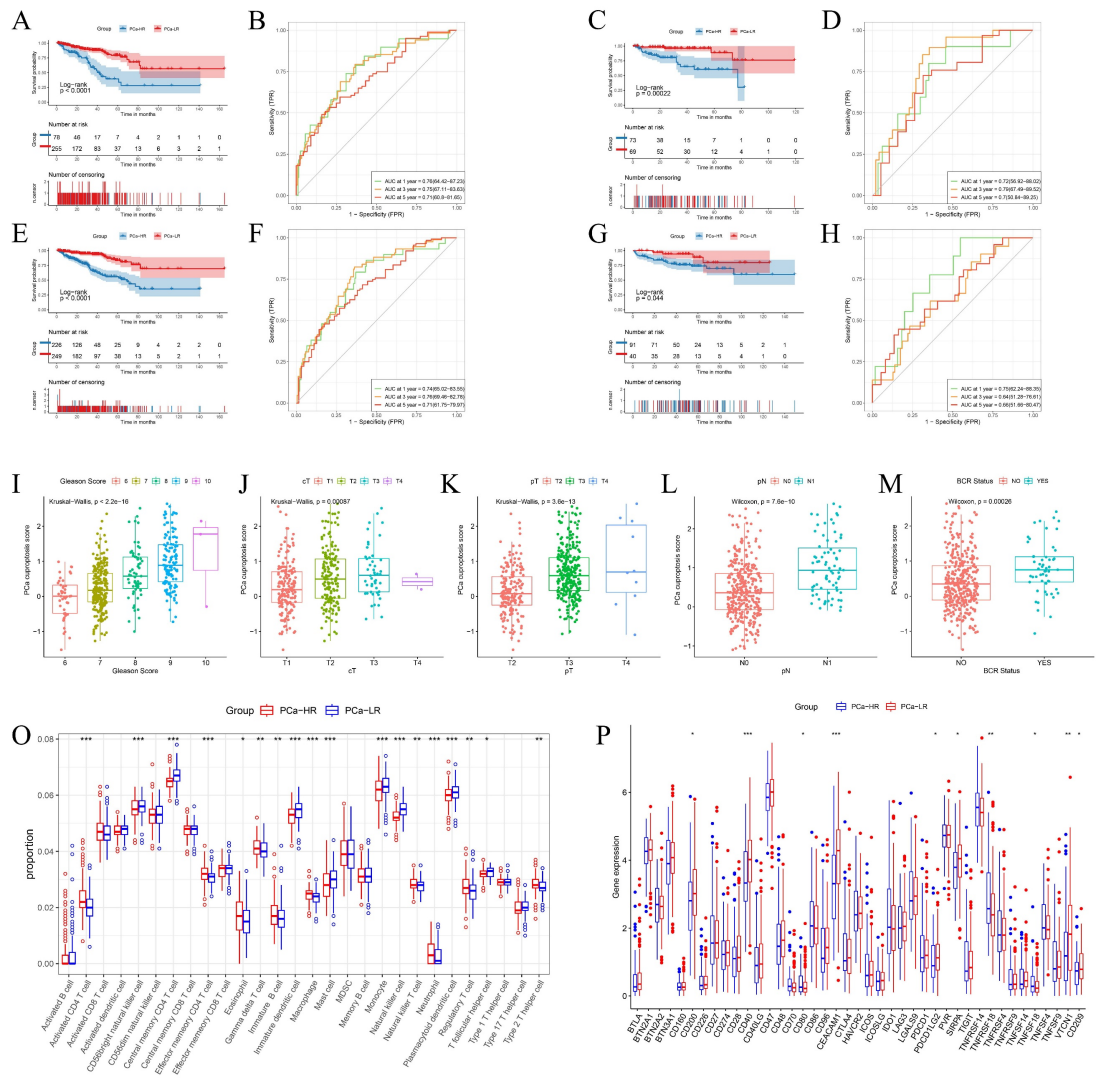
Differences in Prognosis, Clinical Information, and Immune Microenvironment Between PCa-HR and PCa-LR. (A-H) Survival analysis and ROC curves among different risk groups of training set (A-B), internal validation set (C-D), TCGA overall validation set (E-F) and MSKCC validation set (G-H). (I-M) The boxplot shows clinical information such as TNM stage, Gleason score, and BCR status between different risk groups in the TCGA training set. (O) Levels of immune cell infiltration in the PCa-HR and PCa-LR groups. (P) Levels of expression of immune checkpoints in PCa-HR and PCa-LR groups. cT represents clinical T staging. pT represents pathological T staging. pN represents pathological N staging. BCR represents Biochemical recurrence. * p<0.05, ** *p*<0.01, *** *p*<0.001.

To assess the prognosis of patients with different risk stratification, we performed survival analyses of patients in the TCGA training cohort, the TCGA validation cohort, the overall TCGA cohort, and the Memorial Sloan Kettering Cancer Center (MSKCC) external validation cohort. Survival of the PCa-LR group in the TCGA training cohort was significantly better than that of the PCa-HR group (Fig. 4A). This trend was consistent in the TCGA internal validation cohort (Fig. 4C), the overall TCGA validation cohort (Fig. 4E), and the MSKCC external validation cohort (Fig. 4G).

The predictive performance of the model was assessed using the ROC (Receiver Operating Characteristic) curve. The model predicted AUC (Area Under the Curve) values of 0.76, 0.75, and 0.71 for 1-year, 3-year, and 5-year DFS in the TCGA training cohort (Fig. 4B), 0.72, 0.79, and 0.7 for the TCGA validation cohort (Fig. 4D), and 0.74, 0.76, and 0.71 for the overall TCGA cohort (Fig. 4F). In the MSKCC external validation set, the AUC values for 1, 3, and 5 years were 0.75, 0.64, and 0.66, respectively (Fig. 4H). These results collectively suggest that the model has a robust predictive ability.

PCa cuproptosis scores were associated with poorer DFS. Higher PCa cuproptosis scores correlated with higher Gleason scores, later clinical T-staging (cT), pathological T-staging (pT), and pathological N-staging (pN). PCa cuproptosis scores were significantly higher in patients who experienced biochemical recurrence (*p* < 0.05) (Fig. 4I-M). The same trend was observed in the TCGA validation cohort and the overall TCGA cohort.

Our prognostic model, constructed on the basis of cuproptosis immune-related genes (CIRGs), demonstrated favorable performance and stability in predicting DFS in PCa patients. To this end, we further revealed differences in the immune microenvironment and the expression of immune checkpoints in patients with different cuproptosis risk groups to provide new insights for personalized immunotherapy in PCa patients.

We compared the infiltration of immune cells between the PCa-HR and PCa-LR groups and found that the PCa-LR group had a higher proportion of CD56 bright natural killer cells, central memory CD4 T cells, immature dendritic cells, mast cells, monocytes, natural killer cells, plasmacytoid dendritic cells, and T follicular helper cells. Conversely, the PCa-HR group had a higher proportion of activated CD4 T cells, effector memory CD4 T cells, eosinophils, gamma delta T cells, immature B cells, macrophages, natural killer T cells, neutrophils, regulatory T cells, and type 2 T helper cells (Fig. 4O). The expression of immune checkpoint genes in the two groups presented a different landscape (Fig. 4P). Notably, TNFRSF18 expression was significantly upregulated in the PCa-HR group, suggesting it might be a promising target for PCa immunotherapy.

### 2.5 Construction and evaluation of prognostic nomogram

**Figure 5:**
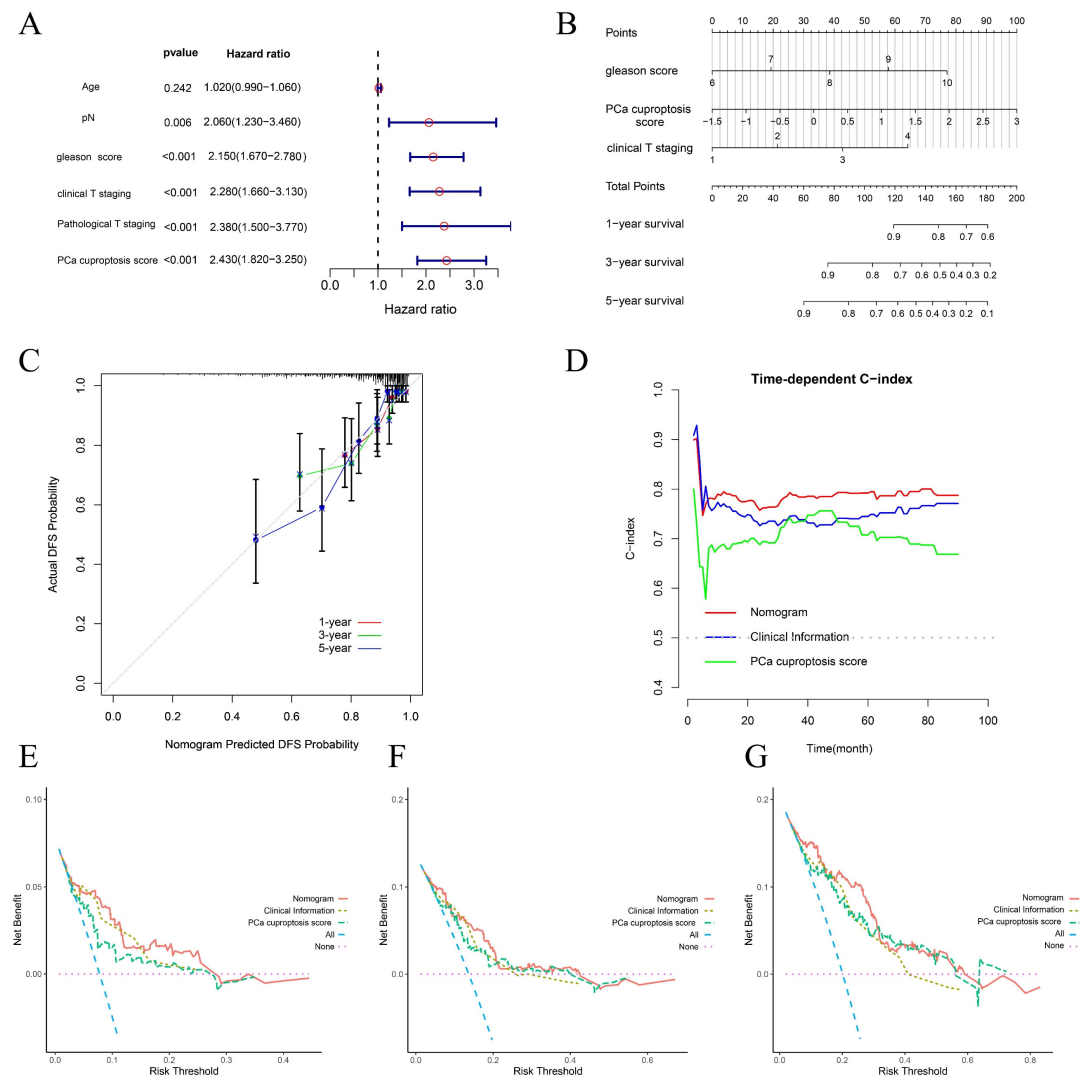
Construction and evaluation of a prognostic nomogram. (A) Forest plot of univariate COX analysis of clinical information and PCa cuproptosis scores. (B) Prognostic nomogram for PCa. (C) Calibration curve for prognostic nomogram. (D) Time-dependent ROC curve for prognostic nomogram. (E-G) Clinical decision curve line for prognostic nomogram. * *p*<0.05, ** *p*<0.01, *** *p*<0.001.

To investigate whether clinical and pathological phenotypes and PCa cuproptosis score could be prognostic factors, age, Gleason score, cT, pT, pN, and PCa cuproptosis score were included in Univariate Cox Regression Analysis. The results indicated that Gleason score, cT, pT, pN, and PCa cuproptosis score were all prognostic factors for PCa DFS (Fig. 5A). We then identified independent predictors of DFS through multifactorial Cox regression analysis, ultimately incorporating Gleason score, cT, pT, pN, and PCa cuproptosis score to construct a nomogram (Fig. 5B). Calibration curves demonstrated that the nomogram's predictions of DFS survival at 1, 3, and 5 years were in good agreement with observed DFS survival (Fig. 5C). As prediction time increased, the concordance index (C-index) of the nomogram stabilized above 0.8, which was higher than that of Gleason score, cT, and PCa cuproptosis score alone, suggesting that the nomogram better predicted DFS at 1, 3, and 5 years in patients with PCa (Fig. 5D). We then performed a decision curve analysis (DCA) to assess clinical benefit. Compared to using only clinical information or the PCa cuproptosis score, the nomogram provided the greatest net benefit over a wider range of thresholds, with the DCA curve performing best in predicting 5-year DFS (Fig. 5E-G).

### 2.6 Differential expression analysis and enrichment analysis between PCa-HR and PCa-LR groups

**Figure 6:**
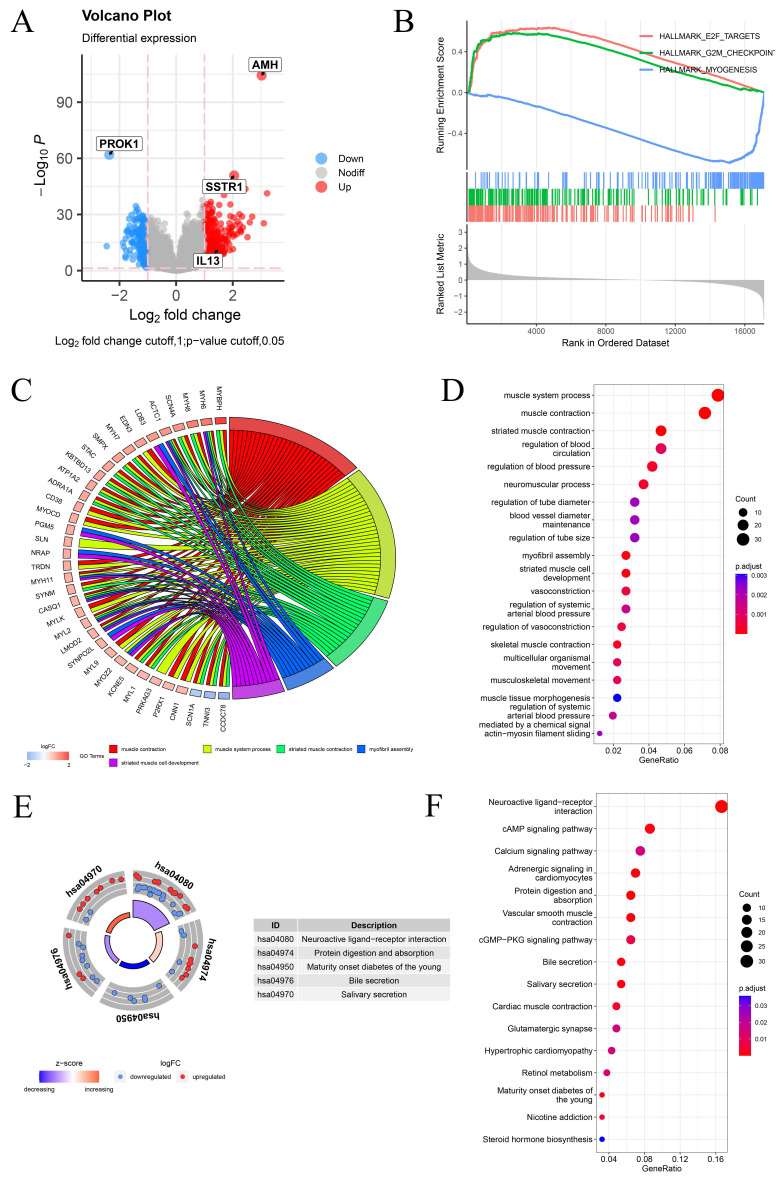
Differential Expression Analysis and Enrichment Analysis Between PCa-HR and PCa-LR Groups. (A) The volcano map shows differentially expressed genes between PCa-HR and PCa-LR groups. (B) GSEA enrichment analysis between PCa-HR and PCa-LR groups. (C-D) GO enrichment analysis of differentially expressed genes between PCa-HR and PCa-LR groups. (E-F) KEGG enrichment analysis of differentially expressed genes between PCa-HR and PCa-LR groups. * p<0.05, ** *p*<0.01, *** *p*<0.001.

Differential expression analysis was conducted to explore potential factors contributing to differences in survival and clinical phenotypes between PCa-HR and PCa-LR groups. Genes with |log2FC| > 1 and *p* < 0.05 were considered differentially expressed between the two groups and visualized using a volcano plot (Fig. 6A). We conducted GSEA enrichment analysis between PCa-HR and PCa-LR groups and identified the three most significant pathways. The aggressive clinical phenotype observed in the PCa-HR group can be mechanistically linked to the significant enrichment of the E2F_TARGETS and G2M_CHECKPOINT pathways. In contrast, the PCa-LR group was characterized by MYOGENESIS pathway enrichment, suggesting that cuproptosis may influence the progression of PCa by modulating these pathways (Fig. 6B). GO enrichment analysis revealed that differentially expressed genes were primarily associated with biological processes such as muscle system processes, muscle contraction, and striated muscle contraction (Fig. 6C-D). KEGG pathway enrichment analysis indicated that differentially expressed genes were predominantly enriched in neuroactive ligand-receptor interaction, cAMP signaling pathway, calcium signaling pathway, and other pathways (Fig. 6E-F).

### 2.7 Building BRFS prognostic models based on multiple machine learning algorithms

**Figure 7:**
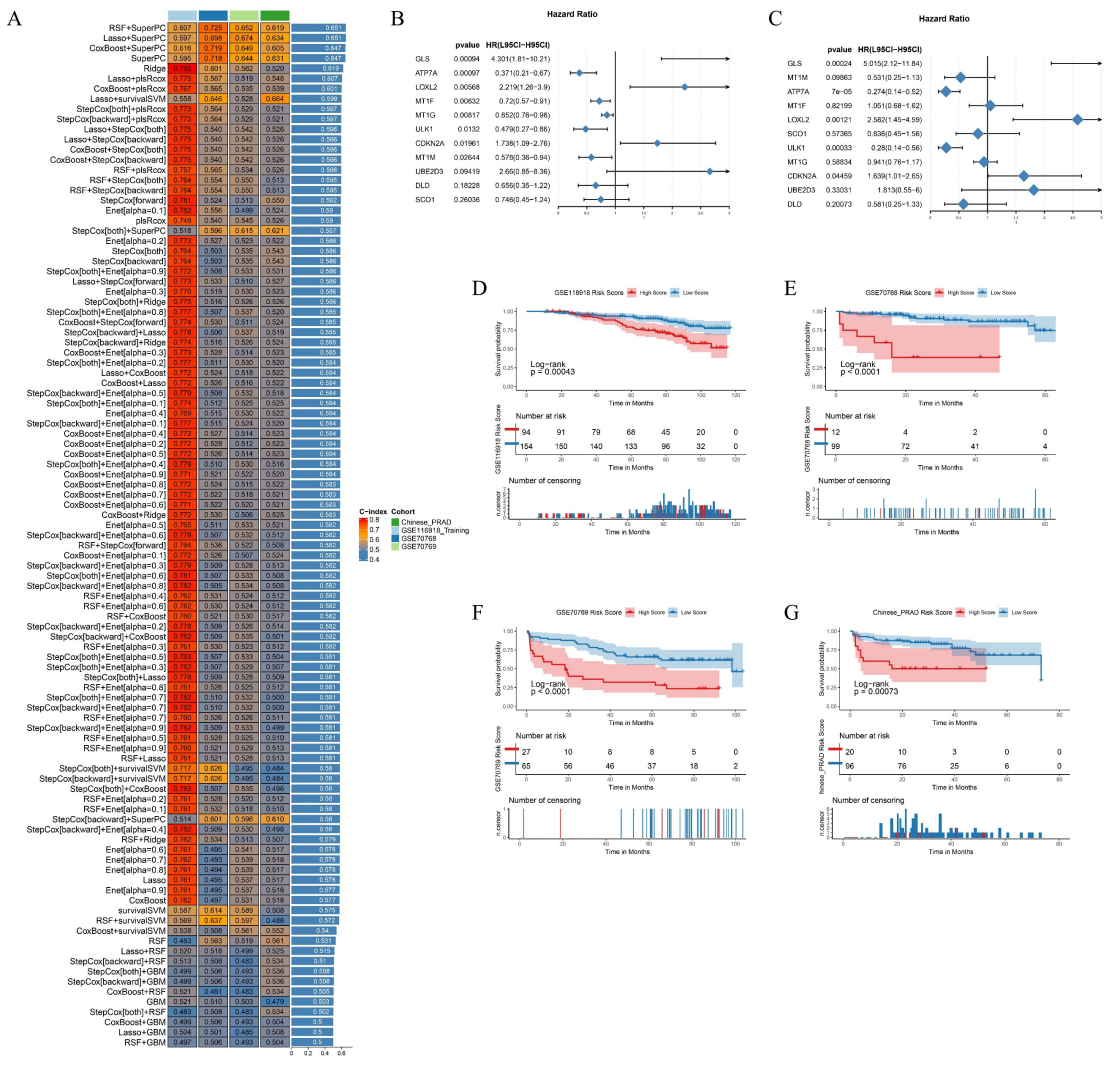
Building BRFS Prognostic Models Based on Multiple Machine Learning Algorithms. (A) Prognostic prediction model for BRFS based on multiple machine learning constructs. (B-C) Forest plot of Univariate Cox Regression Analysis and Multivariate Cox Regression Analysis for genes included in the RSF+SuperPC model. (D-G) Survival curves for training and validation cohorts. * p<0.05, ** *p*<0.01, *** *p*<0.001.

Biochemical recurrence (BCR) is a common indicator of recurrence and distant metastasis in PCa. Early detection and intervention can effectively mitigate these risks. In the TCGA cohort, patients who underwent biochemical recurrence exhibited higher PCa cuproptosis scores, indicating that CRGs may be involved in the biochemical recurrence of PCa. Utilizing the GSE116918 PCa cohort as a training set, we developed a predictive model for biochemical recurrence-free survival (BRFS) based on CRGs and multiple machine learning algorithms. To validate the robustness of the model, we incorporated the Chinese PCa database cohort, the GSE70768 cohort, and the GSE70769 cohort as validation sets. The RSF+SuperPC model exhibited favorable performance in predicting BRFS, achieving a C-index of 0.607 in the GSE116918 training cohort, 0.725 in the GSE70768 validation cohort, 0.652 in the GSE70769 validation cohort, and 0.619 in the Chinese PCa database cohort (Fig. 7A). In the RSF+SuperPC model, GLS (HR=4.301, *p*=0.00094), LOXL2 (HR=2.219, *p*=0.00568), and CDKN2A (HR=1.738, p=0.01961) were identified as risk factors for BRFS. Furthermore, ATP7A (HR=0.371,* p*=0.00097), MT1F (HR=0.72, *p*=0.00632), MT1G (HR=0.777, *p*=0.00777), ULK1 (HR=0.479, *p*=0.0132) and MT1M (HR=0.578, *p*=0.02664) were identified as protective factors for BRFS. GLS (HR=5.015, *p*=0.00024), LOXL2 (HR=2.582, *p*=0.00121), ATP7A (HR=0.274, *p*<0.001), and ULK1 (HR=0.28, *p*=0.00033) were identified as independent prognostic factors for BRFS (Fig. 7B-C). A risk score was calculated for each patient in the training and validation cohorts, with patients classified as high- or low-risk based on the optimal cut-off values. It was observed that patients in the high-risk group exhibited a poorer prognosis compared to those in the low-risk group (Fig. 7D-G).

**Figure 8:**
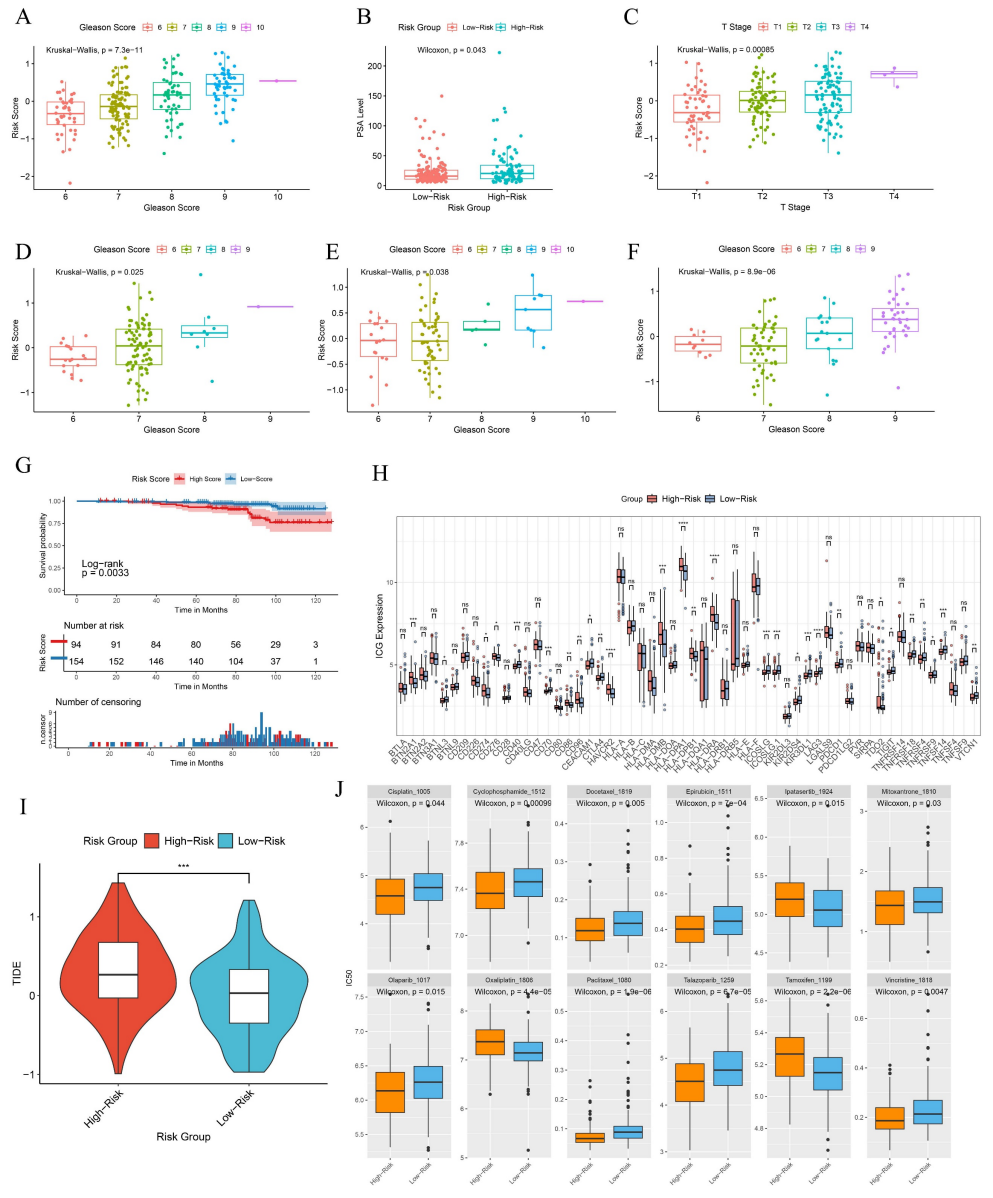
Clinical information and immunophenotypic differences in different model groups. (A-C) Differences in Gleason scores, PSA levels, and clinical T-stages among patients in different risk groups of the GSE116918 cohort. (D-F) Differences in Gleason scores among patients with different risk subgroups in the GSE70768 cohort, GSE70769 cohort and the Chinese PCa Cohort. (G) Prognostic survival curves for MFS in patients with different risk groups. (H) Differences in immune checkpoint gene expression in patients with different risk groups. (I) TIDE scores for patients in different risk subgroups. (J) Prediction of drug sensitivity in patients with different risk groups. * *p*<0.05, ** *p*<0.01, *** *p*<0.001.

In the GSE116918 cohort, patients with high-risk scores exhibited a tendency towards higher Gleason scores, elevated PSA levels and more advanced clinical T stages (Fig. 8A-C). A similar trend was observed in the GSE70768 cohort, the GSE70769 cohort, and the Chinese PCa cohort with respect to Gleason scores, which were found to be statistically significant (Fig. 8D-F). Patients with high-risk scores exhibited a significantly inferior metastasis-free survival (MFS) compared to those in the low-score group (Fig. 8G). This indicates that the BRFS prognostic prediction model is associated with an unfavorable prognosis and a more severe clinical phenotype. Additionally, there is a notable discrepancy in the expression of immune checkpoints (Fig. 8H). Tumors employ two distinct mechanisms to evade immune detection. Firstly, immunosuppressive factors can impede T-cell infiltration. Secondly, despite a high infiltration of cytotoxic T-cells, these T-cells may be functionally inactive. The Tumor Immune Dysfunction and Exclusion (TIDE) algorithm was employed to provide a comprehensive assessment of the two mechanisms of immune escape observed in tumors. The BRFS high-risk group exhibited higher TIDE scores, indicating that patients in this group may have inferior outcomes with immune checkpoint therapy (Fig. 8I). The drug prediction model indicates that patients in the high-risk group have low IC50 values for Ipatasertib, Oxaliplatin, and Tamoxifen, suggesting the potential for benefit from treatment with these drugs. In contrast, patients in the low-risk group may be more sensitive to treatment with Cisplatin, Olaparib, Paclitaxel, Talazoparib, Epirubicin, Cyclophosphamide, Mitoxantrone, Vincristine, and Docetaxel (Fig. 8J).

### 2.8 PROK1 is a promising antioncogene

**Figure 9:**
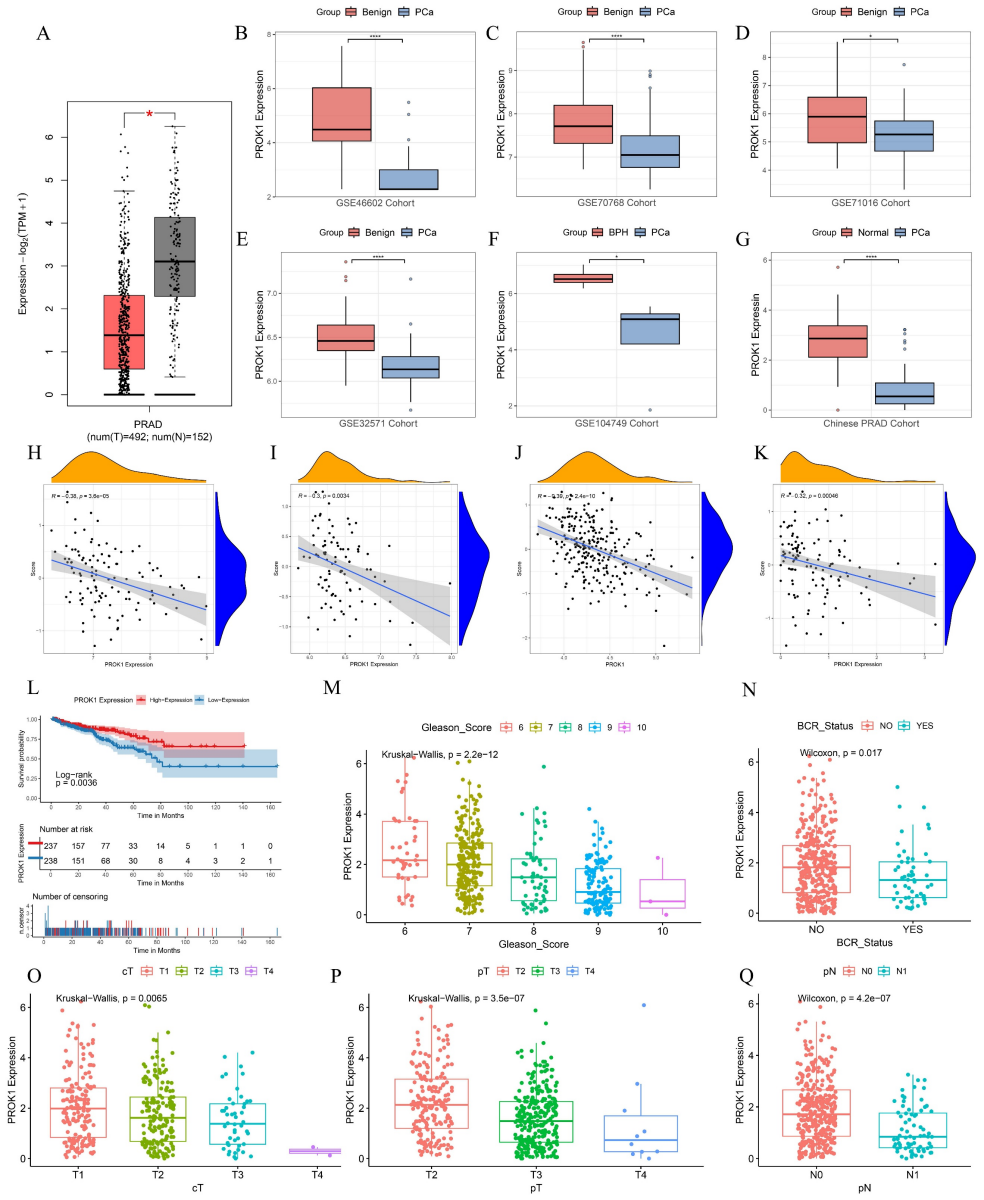
PROK1 is a promising antioncogene. (A-G) PROK1 expression in GEPIA, GSE46602, GSE70768, GSE71016, GSE32571, GSE104749, and Chinese PCa cohorts. (H-K) Scatterplot validating the correlation between PROK1 expression and BRFS risk score in GSE70768 (H), GSE70769 (I), the Chinese PCa cohort (J), and GSE116918 (K). (L) Survival analysis of different PROK1 expression patterns on DFS prognosis. (M-Q) Box plots showing clinical information such as TNM staging, Gleason score, and BCR status between different PROK1 expression groups. * p<0.05, ** *p*<0.01, *** *p*<0.001.

PROK1 exhibited a negative coefficient in the DFS prognostic prediction model formula for PCa, and multifactorial COX regression analysis indicated an HR = 0.754 (*p* < 0.05), suggesting that it may play an oncostatic role. We further validated this in multiple datasets, including GEPIA, GSE46602, GSE70768, GSE71016, GSE32571, GSE104749, and the Chinese PCa cohort, where the results showed that PROK1 expression was significantly downregulated in tumors (Fig. 9A-G). PROK1 expression also demonstrated a significant negative correlation with BRFS scores across multiple cohorts, including GSE70768 (Fig. 9H), GSE70769 (Fig. 9I), the Chinese PCa cohort (Fig. 9J), and GSE116918 (Fig. 9K). Based on median PROK1 expression values, patients in the TCGA cohort were divided into high and low expression groups, and survival analyses suggested that higher PROK1 expression was associated with a better DFS prognosis (Fig. 9L). PROK1 expression decreased with elevated Gleason scores, tumor grade, and BCR status, suggesting that the progression of PCa may be related to the inhibition of PROK1's oncogenic effect (Fig. 9M-Q).

**Figure 10:**
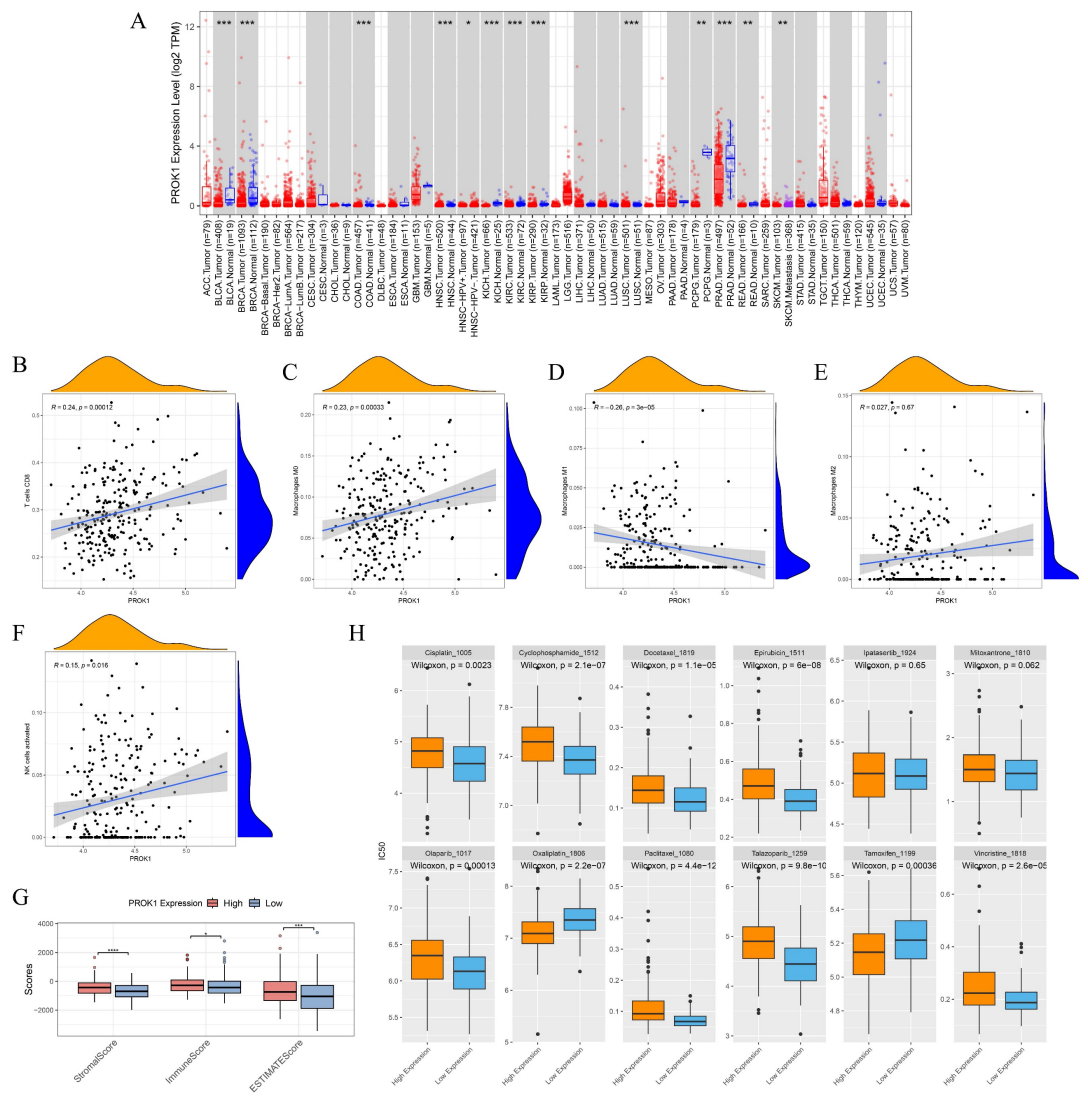
PROK1 is a promising antioncogene. (A) Pan-cancer analysis of PROK1. (B-F) Scatter plot of correlation between PROK1 expression and abundance of immune cell infiltration. (G) Box plot showing the relationship between PROK1 expression and immunity score. (H) Drug sensitivity prediction in patients with different expression subgroups of PROK1. * p<0.05, ** *p*<0.01, *** *p*<0.001.

The expression of PROK1 is different in various tumors, and the expression of PROK1 is down-regulated in PCa (Fig. 10A). PROK1 expression was negatively correlated with the infiltration abundance of CD8 T cells, macrophage M0, macrophage M2, and activated NK cells, while showing a positive correlation with the infiltration abundance of macrophage M1 (Fig. 10B-F). Patients with high PROK1 expression had significantly higher immunity, stromal, and ESTIMATE (Estimation of stromal and immune cells in malignant tumor tissues using expression data) scores, suggesting that PROK1 may modulate the tumor immune microenvironment to exert a cancer-inhibitory effect (Fig. 10G). Drug sensitivity analysis indicated that patients with low PROK1 expression were sensitive to Cisplatin, Cyclophosphamide, Docetaxel, Epirubicin, Olaparib, Paclitaxel, Talazoparib, and Vincristine, whereas patients with high PROK1 expression might be sensitive to Oxaliplatin and Tamoxifen (Fig. 10H).

### 2.9 PROK1 is associated with cuproptosis activity at the transcriptomic level

To further explore the potential link between PROK1 and cuproptosis, we performed additional bioinformatic analyses. First, we calculated a global cuproptosis process activity score for each patient using single-sample gene set enrichment analysis (ssGSEA). We observed a significant positive correlation between PROK1 expression and this cuproptosis activity score (Figure S4), suggesting that higher PROK1 expression is associated with a more active cuproptosis phenotype. Second, we examined the co-expression relationship between PROK1 and the 56 CRGs. Strikingly, PROK1 expression was significantly correlated with 38 out of 56 CRGs, including the core cuproptosis regulator FDX1 and genes encoding lipoylated proteins such as DLAT and DLD (Figure S5-S6). These transcriptomic associations indicate a close relationship between PROK1 and the cuproptosis network, although the precise mechanistic interplay requires further experimental validation.

### 2.10 PROK1 is expressed at low levels in PCa and inhibits PCa progression

**Figure 11:**
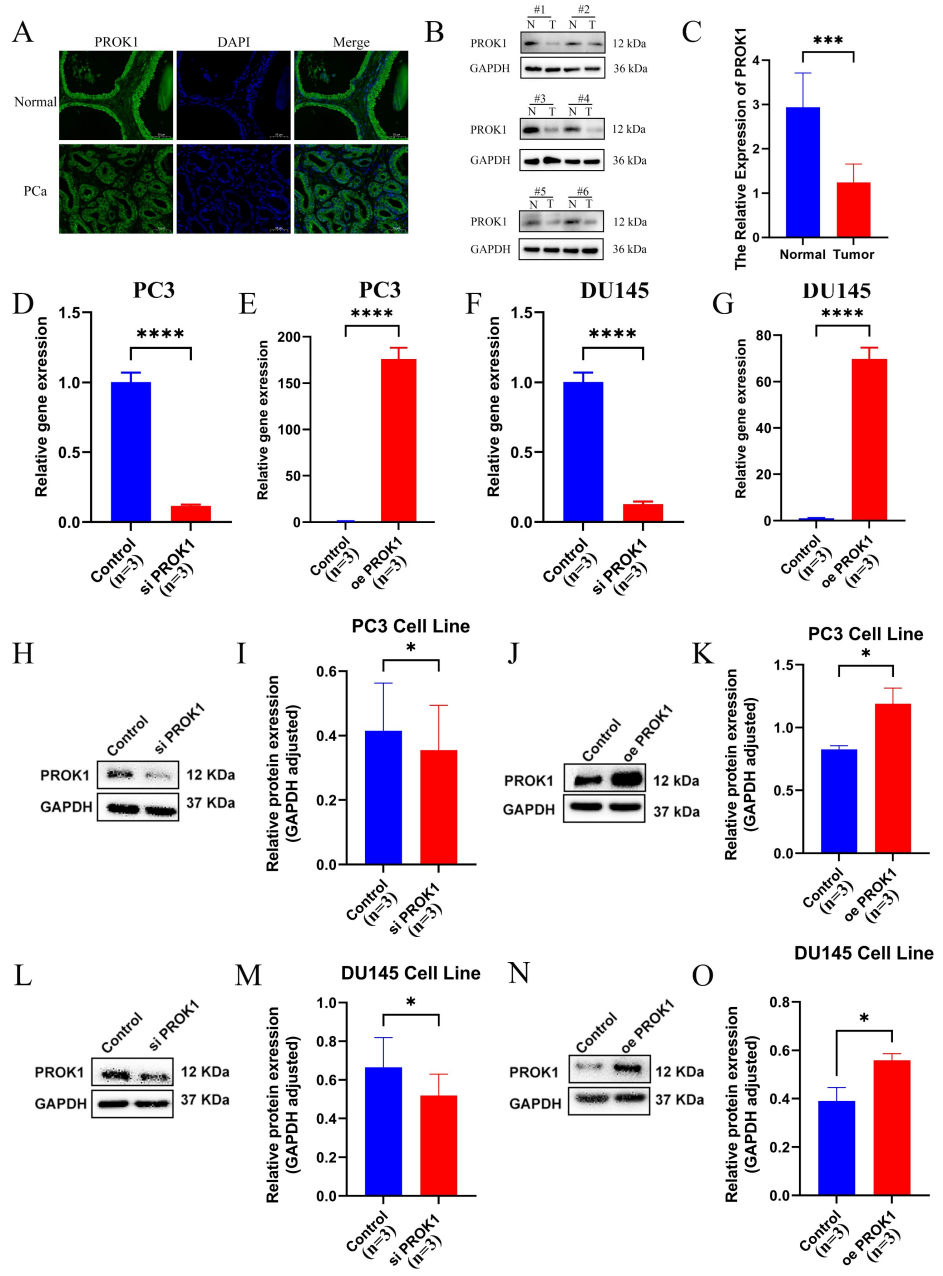
PROK1 inhibits the progression of PCa. (A) Tissue immunofluorescence of PCa and normal prostate tissue. (B-C) Western blot assays of normal prostate cells and PCa cells. (D-G) Real-time Quantitative PCR verified the efficiency of PC3(D-E) and DU145 (F-G) cell lines to knockdown and overexpress PROK1. (H-O) Western blot assays verified changes in PROK1 protein in cell lines after knockdown and overexpression of PROK1. * p<0.05, ** *p*<0.01, *** *p*<0.001.

Tissue samples from clinical PCa and normal prostate were collected, and section fluorescence confirmed that PROK1 expression was lower in PCa than in normal prostate tissue (Fig. 11A). Western blot assays corroborated this finding (Fig. 11B-C). Transfection was performed in the PC3 (Fig. 11D-E) and DU145 (Fig. 11F-G) PCa cell lines to knock down and overexpress PROK1, and the transfection efficiency was verified by real-time quantitative PCR (RT-qPCR). The Western blot assays showed PROK1 expression was elevated in the overexpression group and decreased in the knockdown group (Fig. 11H-O).

**Figure 12:**
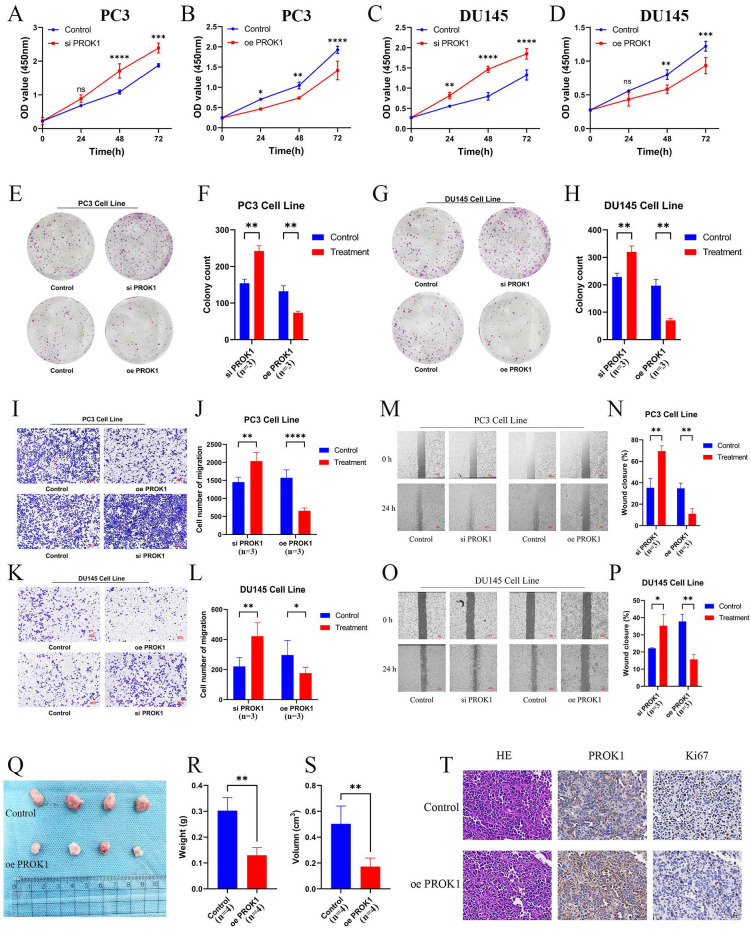
PROK1 inhibits the progression of PCa. (A-D) Proliferation profiles of CCK8 after knockdown and overexpression of PROK1 in PC3 (A-B) and DU145(C-D) cell lines. (E-H) Plate cloning experiments after knockdown and overexpression of PROK1. (I-L) Transwell invasion experiment to validate the invasion ability of PC3 (I-J) and DU145 (K-L) cell lines after knockdown and overexpression of PROK1. (M-P) Migration ability of PC3 (M-N) and DU145 (O-P) cell lines after knockdown and overexpression of PROK1. (Q-S) Differences in weight and volume of subcutaneous tumor formation in nude mice after overexpression of PROK1. (T) Immunohistochemical sections of overexpressed PROK1. * *p*<0.05, ** *p*<0.01, *** *p*<0.001.

CCK-8 and colony formation assays demonstrated that cells in the siPROK1 group proliferated at an accelerated rate, whereas proliferation of cells in the oePROK1 group was slower than that in the control group, showing the same trend in both PC3 (Fig. 12A,B,E,F) and DU145 (Fig. 12C,D,G,H) cell lines. The invasive ability of cells in the siPROK1 group was enhanced, whereas it was decreased in the oePROK1 group, showing similar trends in both PC3 (Fig. 12I,J) and DU145 (Fig. 12K,L) cell lines. The migratory ability of cells in the siPROK1 group was significantly enhanced, while that of cells in the oePROK1 group was significantly reduced compared to the control group (Fig. 12M-P). The xenograft assay was conducted by subcutaneously injecting nude mice with PC3 cells, and it was found that the weight and volume of subcutaneous tumors formed after injection of tumor cells overexpressing PROK1 were smaller than those of the control group (Fig. 12Q-S). The immunohistochemical results indicated higher PROK1 protein expression in the cytoplasm of the oePROK1 group and lower Ki67 signal compared to the control group, suggesting inhibited proliferative activity (Fig. 12T).

To investigate whether PROK1 is involved in the regulation of prostate cancer through the apoptotic pathway, we added copper ions to the culture medium of the PC3 cell line to induce cuproptosis. Subsequently, Western blot analysis was performed on the control group, siPROK1 group, and oePROK1 group to detect the expression levels of cleaved caspase-3, Bax, and Bcl-2 proteins. The experimental results suggested that PROK1 can exert its tumor-suppressive effect by regulating the expression of the aforementioned apoptosis-related proteins and activating the apoptotic pathway (Fig.S3).

Based on these experimental results, we concluded that PROK1 expression in PCa was significantly lower than in normal prostate tissues, and that overexpression of PROK1 significantly inhibited the proliferation, invasion, and migration of PCa cells, suggesting that PROK1 may serve as a promising cancer suppressor.

## 3. Discussion

Copper is a trace element present in the human body and is involved in a number of signaling pathways^28^. In previous studies, copper has been demonstrated to act as a catalytic and structural cofactor of enzymes, thereby playing a role in a number of important biological processes. These processes are involved in a multitude of biological processes that are indispensable for cellular survival, including cell metabolism, signal transduction and energy production^6^. However, an excess of copper in the body can also result in cell death. The role of copper in cancers has been a topic of considerable interest to many scientific researchers. Previous studies have demonstrated that patients with lung, breast, gallbladder, stomach, and thyroid cancers exhibit significantly elevated serum copper levels compared to normal controls^29^-^33^. Additionally, the serum copper concentration is observed to be higher in patients with more advanced clinical stages of lung cancer^34^. A study examining the impact of serum electrolytes on PCa revealed that men with PCa exhibit elevated levels of copper in their serum^35^, although the precise mechanism remains unclear.

We collated the CRGs that had been previously identified through research. It was observed that the majority of CRGs exhibited differential expression. The patients were divided into two groups based on the median CRG expression levels, and the survival comparisons revealed that 16 genes were associated with patient outcomes. A PCa diagnostic model was constructed using CRGs based on multiple machine learning algorithms, with an external validation set used for verification. The results indicated promising diagnostic performance of the model. The results of the cluster analysis of the CRGs were validated by PCA, which revealed significant survival differences between the clusters, along with variations in the clinical phenotypes and immune scores. The GSVA enrichment analysis indicated significant differences in immune gene set enrichment between the groups, thereby suggesting that cuproptosis may exert an influence on the immune microenvironment of PCa. By intersecting CRGs with immune gene sets and applying univariate COX analysis, multivariate COX analysis, and a random forest algorithm, we developed a model for calculating the PCa cuproptosis score. The PCa cuproptosis score was found to correlate with poorer survival, an advanced clinical stage, and a higher Gleason score. The univariate and multivariate COX analyses indicated that the PCa cuproptosis score is a prognostic factor. The score was incorporated into a nomogram with other clinical data, thereby demonstrating its prognostic utility for patients with PCa. The DFS prognostic model scores constructed by CRGs were found to be significantly higher in PCa patients with biochemical recurrence than in those without. A BRFS prognostic model based on CRGs was constructed using multiple machine learning algorithms and validated in other GEO cohorts and a Chinese PCa cohort, thereby demonstrating the robustness of the model. Higher risk scores were predictive of worse BRFS, higher Gleason scores and clinical staging. Furthermore, we predicted the immunotherapy response in patients with different risk subgroups and screened them for potentially effective drugs, thereby suggesting potential avenues for the personalized treatment of PCa patients in the future.

This finding is well supported by existing literature that positions these pathways as central drivers of tumor aggressiveness. The E2F family of transcription factors, particularly E2F1 and E2F7 which are often upregulated in tumors, are master regulators of cell cycle progression and are known to enhance tumor cell proliferation, angiogenesis, and invasiveness^36^. Similarly, the G2M_CHECKPOINT is a key regulator of cell division, and its heightened activity is an established marker of clinical aggressiveness across cancer types, correlating with increased invasiveness, metastatic potential, and worse patient survival. For example, pancreatic cancer patients with high G2M scores demonstrate worse outcomes, particularly following resection with positive margins. Additionally, breast tumors displaying high activity of the G2M checkpoint pathway exhibit increased invasiveness and metastatic potential. Elevated scores of this pathway correlate with clinical aggressiveness, patient survival rates, and adverse prognosis in metastatic lesions of ER-positive/HER2-negative breast cancer^37^, ^38^. The PCa-LR group showed significant enrichment of the MYOGENESIS pathway. Currently, there are relatively few studies on the specific mechanism of action of this pathway in tumors. Some research suggests that muscle precursor cells (MPCs) contained in skeletal muscle can regenerate muscle fibers and be used for the treatment of urinary incontinence, including in patients who have undergone prostatectomy. Moreover, the application of MPCs in the vicinity of prostate cancer can reduce tumor growth and has been shown to inhibit the formation of metastases^39^. Therefore, we propose that the coordinated activation of the E2F_TARGETS and G2M_CHECKPOINT pathways constitutes a core molecular mechanism that drives the aggressive clinical features—such as higher Gleason scores, advanced staging, and poorer survival—which define the PCa-HR subgroup.

Two types of pro-angiogenic factors have been identified to date: vascular endothelial growth factor (VEGF) and tissue-specific proangiogenic factors, such as endocrine gland-derived vascular endothelial growth factor (EG-VEGF), which is also known as PROK1. Previous studies have suggested that PROK1 can function as an oncogene in certain contexts. For instance, it has been reported to be highly expressed in pancreatic cancer (PC) and to promote proliferation and invasion^24^, ^25^, ^40^. Similarly, its expression has been associated with advanced disease and hematogenous metastasis in colon cancer and with advanced stages in ovarian cancer^27^, ^41^. However, our pan-cancer expression analysis presents a more complex and context-dependent picture. While PROK1 did not show significant differential expression in pancreatic cancer, we observed that it was significantly downregulated in tumors from colon cancer (COAD) and ovarian cancer (OV) patients compared to their corresponding normal tissues (Figure 10A). This finding indicates that the role of PROK1 is not universally oncogenic and may vary considerably across different tumor types or specific pathological stages.

Most notably, and in stark contrast to its reported role in other malignancies, our study robustly confirms that PROK1 is expressed at low levels in PCa tissues and functions as a tumor suppressor. Through gain-of-function and loss-of-function experiments in PCa cell lines, we demonstrated that elevated PROK1 expression is associated with reduced growth, invasion, and migration capabilities, whereas its inhibition enhanced these aggressive behaviors.

The precise mechanism underlying the tissue-specific silencing of PROK1 in PCa warrants further investigation. Our exploratory analyses provide initial clues, suggesting a complex regulatory process that may involve transcriptional repression within the unique prostate microenvironment, potentially mediated by factors such as the androgen receptor (AR) signaling axis, or epigenetic silencing via promoter hypermethylation. Elucidating the dominant mechanism—whether it be AR-mediated repression, epigenetic modification, or a combination of both—represents a crucial and compelling direction for our future research.

Collectively, these observations reveal a dual nature of PROK1 in cancer biology. It appears to exert tissue-specific and potentially stage-specific functions, acting as an oncogene in some contexts but as a protective tumor suppressor in prostate cancer. This critical distinction underscores the importance of the cellular and molecular microenvironment in determining PROK1's ultimate functional outcome and highlights its potential as a novel therapeutic target specifically for prostate cancer.

Our study also opens up several promising avenues for future research. First, the exact molecular mechanism behind the tissue-specific suppression of PROK1 in PCa, potentially involving AR signaling or epigenetic regulation, needs to be elucidated. Second, as noted above, the functional connection between PROK1 and the core cuproptosis pathway requires direct experimental validation, for instance, by examining whether PROK1 modulates FDX1 protein levels or the lipoylation status of key enzymes like DLAT.

## 4. Methods

### 4.1 Data acquisition and processing

Gene expression profiles and clinical data of PCa patients were retrieved from The Cancer Genome Atlas (TCGA) (https://portal.gdc.cancer.gov/) and the cBioPortal website (https://www.cbioportal.org/). The inclusion and exclusion criteria were as follows: (1) Both expression data and clinical information had to be available, (2) PCa samples from the primary site were selected, and (3) patients lacking survival information or with survival of less than one month were excluded to ensure study accuracy. In total, 475 PCa patients were included in the study. After acquiring the gene read count expression values of PCa patients, we converted the data to TPM format to ensure comparability with microarray data. In addition, we downloaded gene expression profiles and clinical information for PCa study cohorts GSE116918, GSE70768, GSE70769, GSE104749, GSE32571, GSE3325, GSE46602, and GSE8511 via the Gene Expression Omnibus (GEO) (https://www.ncbi.nlm.nih.gov/geo/). Genes not expressed in the majority of patients were excluded.

Sixty-two CRGs were identified from previous studies^42^-^45^. After excluding genes without expression data in the TCGA dataset, 56 were included in the final analysis.

### 4.2 Construction of PPI network

The interactions of CRGs were initially explored using the STRING database (http://string-db.org), and the protein interactions were subsequently visualized according to degree value in Cytoscape software^46^.

### 4.3 Clustering consensus

Consensus clustering, an unsupervised clustering method, was performed using the R package *ConsensusClusterPlus*, selecting the optimal k value (from 2 to 9) based on the CDF curve^47^.

### 4.4 GSVA

GSVA is an unsupervised gene set enrichment method that can detect subtle differences in pathway activity within a sample population^48^. Immune-related gene sets were retrieved from the MsigDB database (http://www.gsea-msigdb.org/gsea/) and the top 20 GSVA results were visualized in a heatmap.

### 4.5 Acquisition of immune genes associated with cuproptosis

Given the significant survival differences and variations in immune scores and gene sets between the two cuproptosis-related clusters, *DESeq2* R was used to screen for differentially expressed genes between the clusters^49^. Genes with |log2FC| > 1 and p < 0.05 were classified as differentially expressed genes (DEGs). A total of 1,793 immune-related genes were retrieved from the ImmPort database (https://www.immport.org/home)^50^. Overlapping genes between the DEGs and immune-related genes were identified as CIRGs.

### 4.6 Prognostic related signature

The* Caret* R package was used to partition the TCGA data into training and internal verification sets, with a 7:3 ratio. For the CIRGs in the training set, we first performed a univariate Cox regression analysis. To ensure a comprehensive screening of potential prognostic biomarkers while maintaining methodological rigor, we employed a two-stage variable selection strategy. In the initial univariate Cox regression analysis, a relaxed significance threshold (p < 0.2) was applied. This approach is a well-established method to minimize the risk of overlooking genes that, while not meeting the conventional 0.05 significance level in univariate analysis, may contribute valuable predictive power in conjunction with other variables in a multivariate model^51^, ^52^. Subsequently, more stringent machine learning algorithms (random forest) and multivariate Cox regression with stepwise selection based on the Akaike Information Criterion (AIC) were employed to refine the model and prevent overfitting, ensuring the final signature's robustness. Based on the *randomForestSRC* R package, we used random forest algorithm to further screen genes to select the most important genes. We set up 1000 decision trees and selected key genes with a cut-off value of 0.3 for variable relative importance. R package *My.stepwise* was used to further screen CIRGs based on AIC minimum criterion, and the final genes were analyzed by multivariate COX regression analysis. We then constructed prognostic signatures based on four genes, including AMH, PROK1, IL13, and SSTR1. Based on the following calculation formula, we obtained the PCa cuproptosis score of each patient in TCGA cohort.



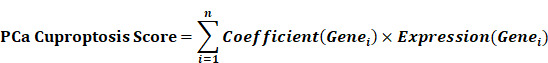



We used the surv_cutpoint function of the survival R package to determine the best cutoff value for dividing risk groups. The TCGA training set, the TCGA internal validation set and the whole TCGA validation set were divided into PCa high-risk group (PCa-HR group) and PCa low-risk group (PCa-LR group). Similarly, the PCa cuproptosis score for the MSKCC external validation set was calculated and divided into PCa-HR group and PCa-LR group.

To evaluate the prognostic significance of signature for PCa, we performed survival analysis for PCa-HR and PCa-LR groups in the validation set and training set based on survival R package. Meanwhile, we plotted the ROC curve and calculate the area under the curve as an assessment of the accuracy of the model based on timeROC R package in TCGA cohort. The superiority of the model was also demonstrated by significant differences in clinical or pathological information between the groups.

### 4.7 Analysis of enrichment

We retrieved and downloaded 50 hallmark gene sets from MSigDB database. Differential expression analysis was performed between the PCa-HR and PCa-LR groups, and the GSEA algorithm was performed using the GSEA function after sorting according to the fold change^53^. false discovery rate (FDR) < 0.25, p < 0.05 and |normalized enrichment score (NES)| > 1 were considered statistically significant. The clusterProfiler R package has powerful gene annotation and enrichment analysis functions, and we used it for GO and KEGG enrichment analyses.

### 4.8 Prognostic related nomogram

In the training set, we used PCa cuproptosis score calculated by the signature as a new parameter to perform univariate COX regression analysis with other clinical or pathological phenotypes. Then stepwise regression analysis was performed, and finally, multivariate COX regression analysis was carried out for the obtained variables. We then drew a nomogram based on a multivariable Cox regression model using the rms R package. Then we calculated the C-index using the pec R package and evaluated the differentiation of nomogram models. A calibration curve was then drawn to evaluate the agreement of the model's predictions with actual observations. We also performed DCA curve to evaluate the clinical applicability of this nomogram model.

### 4.9 Construction of a diagnostic model

The expression data from the training cohort was trained by incorporating 101 combinations of various machine learning algorithms, including Lasso, SVM, glmBoost, Ridge, Enet, Stepglm, RF, plsRglm, and GBM. The resulting models were then validated using the training model on an external cohort, and the average AUC value was calculated for each model.

### 4.10 Construction of a prognostic model for biochemical recurrence survival

Gene expression data from the training cohort was incorporated into 101 combinations of several machine learning algorithms, including Lasso, StepCox, survivalSVM, CoxBoost, Ridge, SuperPC, and others, for training and validation by multiple independent external cohorts. The average C-index was calculated for each model.

### 4.11 Cell culture

PCa cell line PC3 and human normal prostate epithelial cell line RWPE-1 were purchased from Institute of Biochemistry and Cell Biology, CAS (IBCB, Shanghai). All cells were cultured using RPMI-1640 medium containing 10% fetal bovine serum. The incubator was maintained at a constant temperature of 37 °C and contained 5% carbon dioxide.

### 4.12 Cell transfection

The siRNA and overexpression plasmids we used were purchased from Jiman biotechnology company, and configured to the appropriate concentration using DEPC water according to the manufacturer's instructions. When the cell fusion reached 60%, we transfected the cells using Lipofectamine 2000 (Invitrogen, USA).

### 4.13 Quantitative real-time PCR

We extracted total RNA from the cells using TRIzol reagent and then measured the concentration before reverse transcribing it into cDNA using PrimeScript™ RT reagent Kit (Takara, Japan). The primer (Sangon Biotech, China) sequences used are shown in the table below.

### 4.14 Western blot

After washing the cells with PBS, we lysed them using RIPA lysis buffer (Servicebio, China), centrifuged the supernatant and then measured the protein concentration using the BCA method. The proteins were denatured by adding the corresponding volume of 2×loading buffer followed by boiling water for 15 min. After electrophoresis, the membrane was transferred to NC membrane and sealed with skimmed milk, then incubated with primary (ABclonal, China) and secondary antibodies, and finally the bands were detected by fluorescence chemistry.

### 4.15 CCK-8 assay

We selected cells in the logarithmic growth phase and inoculated them in 96-well plates at a density of 2000 per well. CCK-8 solution (Dojindo, Japan) was added at 0 h, 24 h and 48 h and incubated in the incubator for 2 hours, and then the absorbance at 450 nm was measured using a microplate reader (Thermo Fisher Scientific).

### 4.16 Transwell assay

We selected well-grown cells and calculated the concentration. The transwell chambers were lined with Matrigel gel according to the manufacturer's instructions. We added serum-free medium to the upper chamber and medium containing 10% fetal bovine serum to the lower chamber. Then 4×10^4^ cells were added to the upper chamber. The chambers were removed after 24h of incubation, stained with paraformaldehyde and crystal violet, observed under the microscope and the number of cells passing through the chambers was counted.

### 4.17 Cell scratch assay

We cultured the cells in 6-well plates. When the fusion reached 100%, we made equal width scratches in the petri dishes according to the drawn markings. After washing the detached cells using PBS, we added serum-free medium. The results were observed and recorded under microscope at 0h and 24h.

### 4.18 Colony formation assay

800 PCa cells were uniformly inoculated in 12-well plates and cultured for about 2 weeks, with medium changed every 4 days. After the formation of cell clusters, the cells were fixed with 4% paraformaldehyde for 15 min and stained with crystal violet for 30 min.

### 4.19 Hematoxylin and eosin (HE)

Paraffin was removed from the tissue sections on the slides with xylene. Hydrate the tissue sections by passing them through water at a reduced alcohol concentration. The nuclei were stained blue with hematoxylin. Wash the sections to remove excess hematoxylin. Stain the cytoplasm pink with eosin. Increase the alcohol concentration to dehydrate the tissue. Clean the tissue with xylene and photograph it.

### 4.20 Immunohistochemistry (IHC)

The sections were deparaffinized, rehydrated, and subjected to antigen retrieval. Following the blocking of non-specific binding, the primary antibodies (ABclonal, China) were applied, followed by the application of a biotinylated secondary antibody. The signal was developed using a DAB substrate, which produced a brown stain indicative of positive expression. Sections were then counterstained with hematoxylin, dehydrated, and mounted for microscopic evaluation.

### 4.21 Xenograft assay

The male BALB/c nude mice, approximately four weeks old and weighing around 16 g each, were procured from Beijing HFK Bioscience Co. Ltd (Beijing, China). The mice were randomly divided into a control group and an oePROK1 group. After 2 days of acclimatization under specific pathogen-free (SPF) conditions, all mice were subjected to the next stage of the experiment. Subsequently, 2 × 10^6^ of PC3 cells suspended in 100 μl of PBS and PC3 cells of oePROK1 were injected into the axilla of the forelimb of each mouse. Approximately 4 weeks later, all tumors were excised for body weight measurement and immunohistochemical analysis. The animal experiments were approved by the Ethics Committee of Tongji Hospital, Tongji University, Shanghai. All procedures were conducted in accordance with the ethical guidelines set forth to ensure the welfare of the animals.

### 4.22 Statistical analysis

We used R software (version 4.1.3) and GraphPad Prism (version 9.5) for statistical analysis and visualization. For comparison of differences between the two groups, the t test was used for data conforming to homogeneity of variance and normal distribution, otherwise the Wilcoxon rank sum test was used. Kruskal-Wallis test was used to compare differences between three or more groups. Spearman's correlation analysis is used to calculate correlations. Data were presented as mean ± SD. *p* < 0.05 was considered statistically significant.

## 5. Conclusion

This study examined the expression and mutational landscape of cuproptosis genes in PCa, identifying a potential role in regulating tumor immune microenvironments. Based on multiple PCa study cohorts and using a machine learning approach, we constructed diagnostic models for PCa, a BCR prognostic prediction model for PCa, and a DFS prognostic prediction model, and validated their reliability. The tumor-suppressive role of the key gene PROK1 was experimentally confirmed. Our data demonstrate that this effect is primarily mediated through the induction of apoptosis, which can be modulated by copper stress. Although transcriptomic analyses associate PROK1 with cuproptosis activity, direct mechanistic involvement in the core cuproptosis pathway remains to be proven. This work may provide a novel therapeutic target and a foundation for future research into PCa treatment.

## Supplementary Material

Supplementary figures.

## Figures and Tables

**Table 1:** Primer sequences

	Forward primer 5'-3'	Reverse primer 5'-3'
GAPDH	TGACTTCAACAGCGACACCCA	CACCCTGTTGCTGTAGCCAAA
PROK1	AACTGTGTCTGACTGTGCTGTGATC	GCAAGCAAGGACAGGTGTGGTG
