# Supplementary material for: Cuproptosis-related gene PROK1 predicts the diagnosis and prognosis of prostate cancer based on multiple machine learning
Source: J Cancer. 2026 Jan 1;17(2):268–89. doi: 10.7150/jca.113505 (PMC12825426; doi:10.7150/jca.113505)
Supplement: Supplementary file 1 — Supplementary figures. [file jcav17p0268s1.pdf]

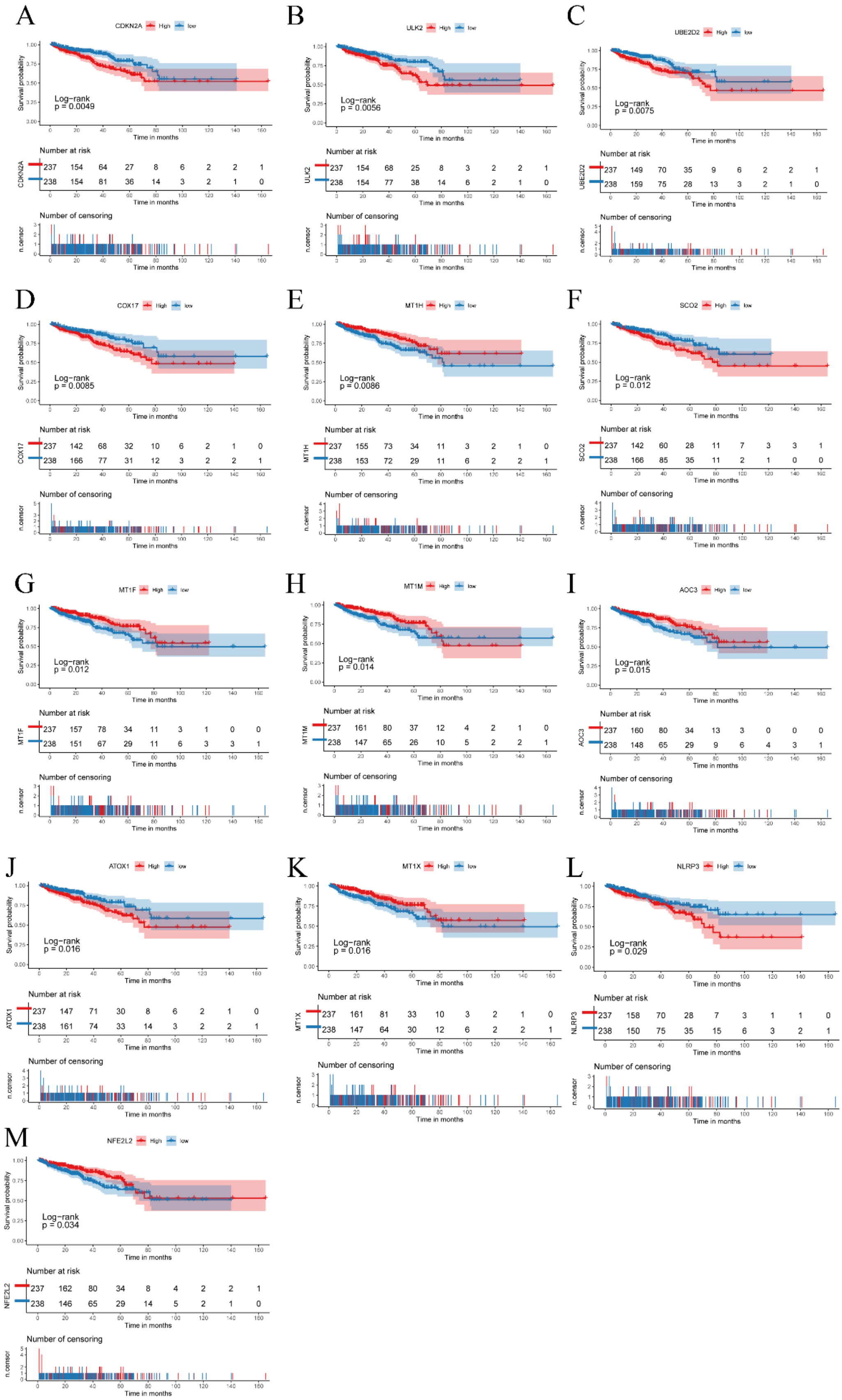

Figure S1. Survival analysis of cuproptosis-related genes. (A−M) Kaplan−Meier curves

showing the impact of cuproptosis-related gene expression on the prognosis of PCa.

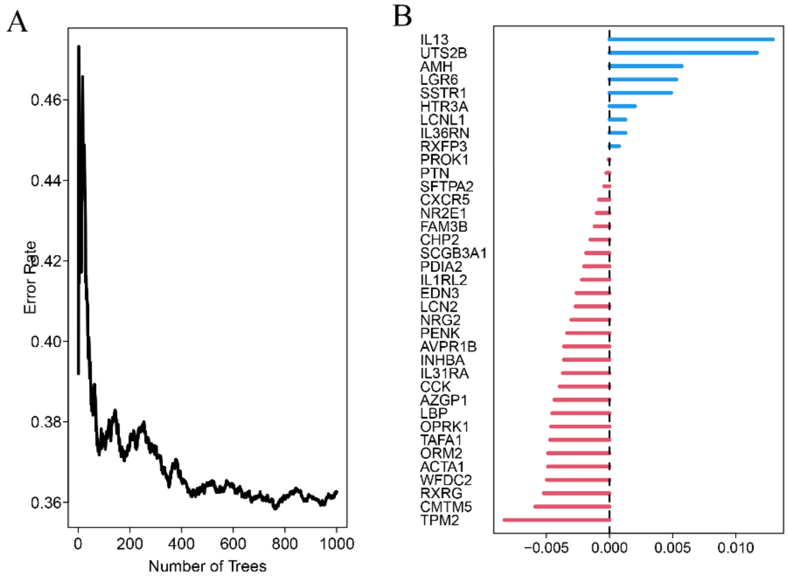

Figure S2. Random forest analysis. (A) OOB (out-of-bag) error rate plotted against the number of trees. (B) Variable importance plot showing the contribution of each gene to the model.

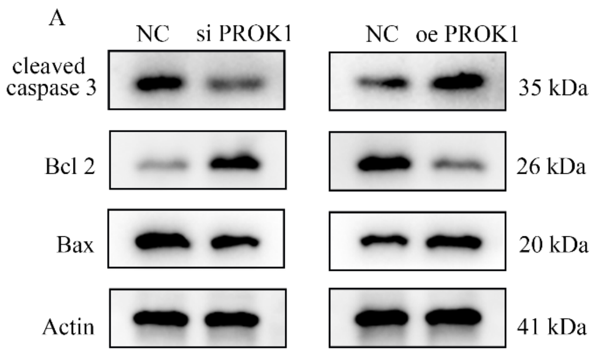

Figure S3. Western blot analysis of apoptosis-related proteins following PROK1 knockdown or overexpression. (A) Representative blots showing cleaved caspase-3, Bcl-2, and Bax expression in NC vs si-PROK1 and NC vs oe-PROK1 groups, with Actin as the loading control.

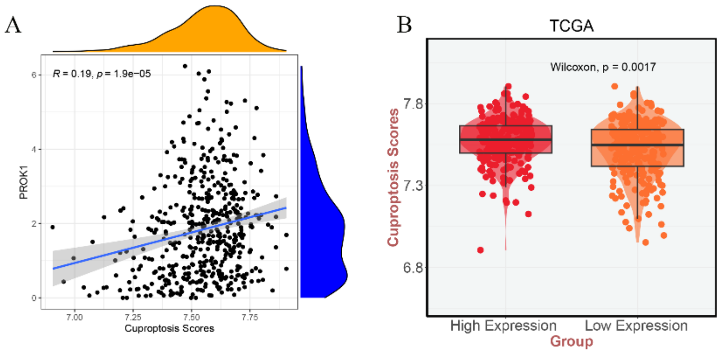

Figure S4. Association between PROK1 expression and cuproptosis in the TCGA cohort. (A) Correlation analysis between PROK1 expression and cuproptosis scores. (B) Comparison of cuproptosis scores between the high- and low-PROK1 expression groups.

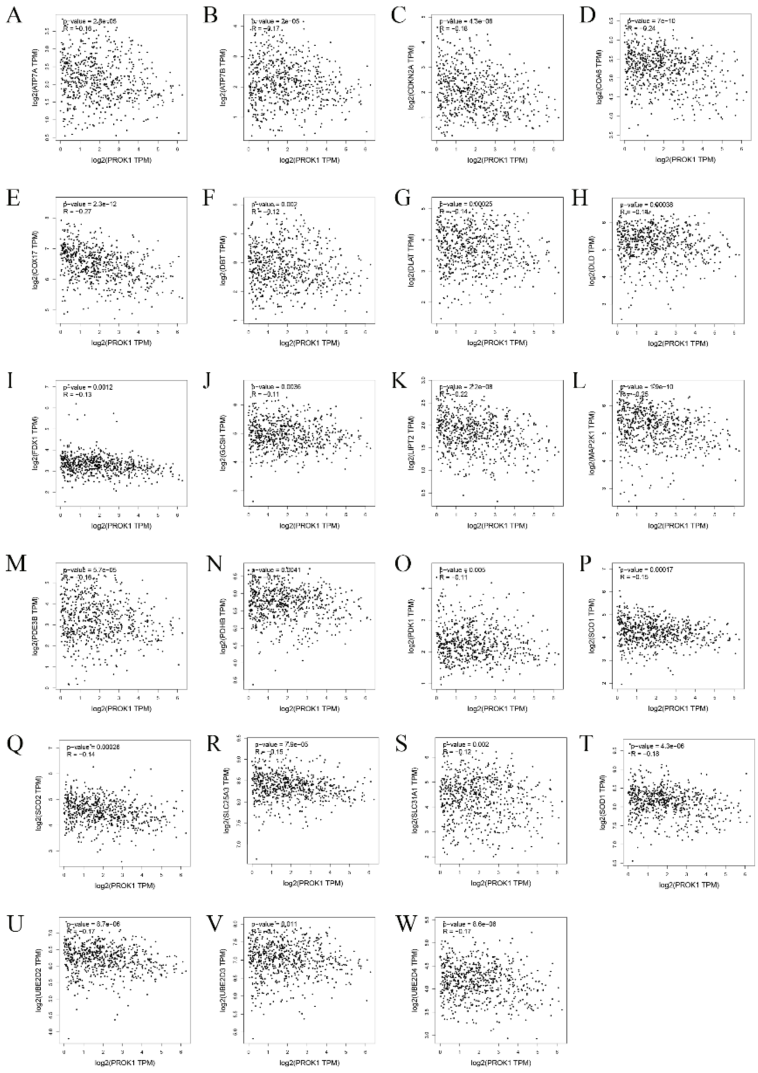

Figure S5. Correlation between PROK1 and cuproptosis-related genes. (A−W) Scatter plots showing significant negative correlations between PROK1 expression and the expression of cuproptosis-related genes.

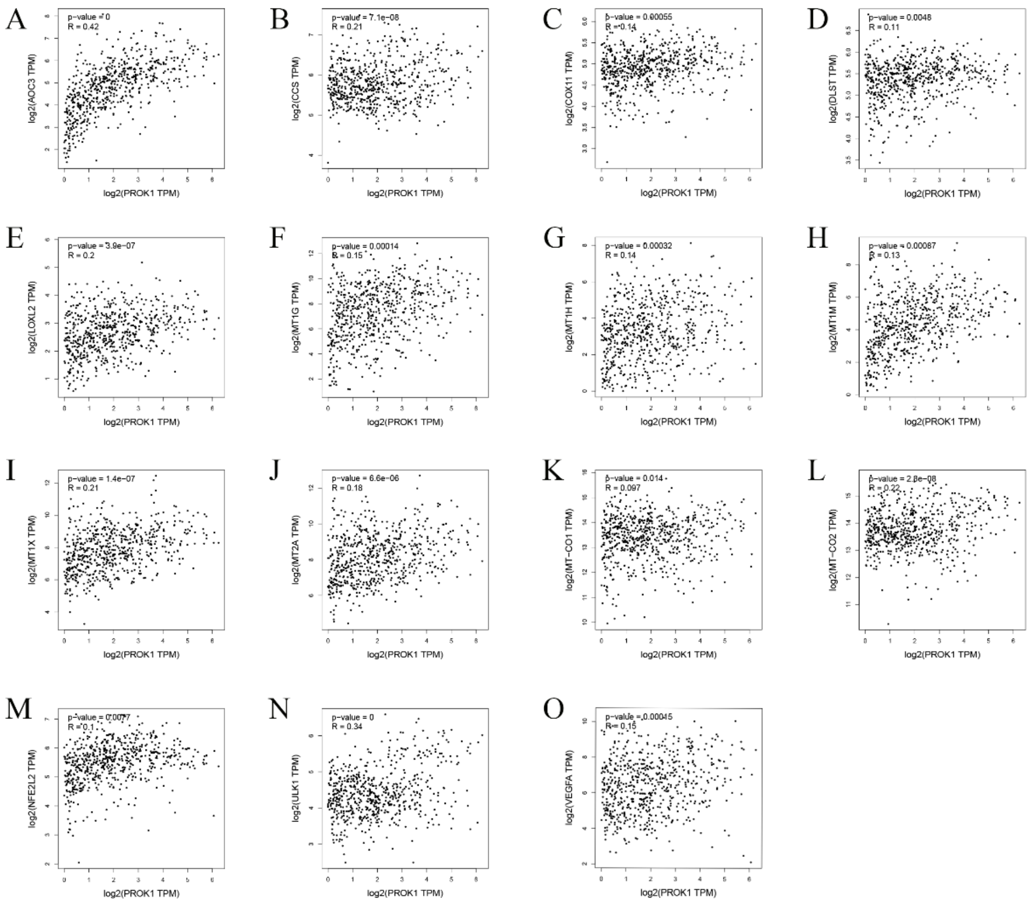

Figure S6. Correlation between PROK1 and cuproptosis-related genes. (A–O) Scatter plots showing significant positive correlations between PROK1 expression and the expression of cuproptosis-related genes.
